# Iron overload disrupts bone homeostasis via dual pathways: inducing ferroptosis of preosteoblastic cells and shaping a proinflammatory immune milieu

**DOI:** 10.3389/fphar.2026.1905654

**Published:** 2026-10-16

**Authors:** Yu Pan, Renfeng Zhao, Chengdong Zhang, Feng Shi, Hui Wu, Dongqin Xiao

**Affiliations:** 1 Research Institute of Tissue Engineering and Stem Cells, School of Basic Medical Sciences and Forensic Medicine, The Second Clinical College of North Sichuan Medical College, Nanchong, Sichuan, China; 2 Department of Clinical Laboratory, Beijing Anzhen Nanchong Hospital of Capital Medical University & Nanchong Central Hospital, Nanchong, Sichuan, China; 3 Collaborative Innovation Center for Tissue Repair Material Engineering Technology, China West Normal University, Nanchong, Sichuan, China; 4 Department of Orthopedics, Beijing Anzhen Nanchong Hospital of Capital Medical University & Nanchong Central Hospital, Nanchong, Sichuan, China

**Keywords:** bone metabolism, ferroptosis, IL-17 pathway, iron overload, osteoimmunology, osteoporosis

## Abstract

Iron overload is regarded as an independent risk factor for osteoporosis, yet the underlying molecular mechanisms remain incompletely understood. In this study, we systematically investigated the impact of iron overload on bone homeostasis using a combination of *in vivo* and *in vitro* models. An iron overload rat model was established, and investigations were complemented by cellular assays utilizing mouse embryonic osteoblast precursor cells (MC3T3-E1) and macrophages. The *in vivo* results demonstrated that iron overload significantly reduced bone mineral density (BMD) and disrupted bone microarchitecture. These structural changes were accompanied by an enhanced inflammatory response within the bone tissue and the aberrant expression of ferroptosis markers. The *in vitro* experiments confirmed that iron overload induced ferroptosis and inhibited osteogenic differentiation in MC3T3-E1 cells by triggering oxidative stress, lipid peroxidation, and mitochondrial damage. This process was closely associated with the significant upregulation of the ATPase H+ transporting V0 subunit D2 (*Atp6v0d2*) gene. Notably, plasmid-mediated knockdown of *Atp6v0d2* substantially alleviated ferroptosis and rescued osteogenic function in iron-overloaded preosteoblastic cells. Furthermore, our study revealed that iron overload drove macrophages toward a pro-inflammatory M1 phenotype. Conditioned medium from these polarized M1 macrophages induced apoptosis and cell cycle arrest in preosteoblastic cells while promoting osteoclast differentiation, an effect mediated by the activation of the IL-17 signaling pathway. In conclusion, this study revealed a dual mechanism by which iron overload disrupted bone homeostasis: it directly induced ferroptosis in osteogenic cells and indirectly shaped a pro-inflammatory immune microenvironment. These findings provide novel insights into the pathogenesis of iron overload-induced osteoporosis and offer a new perspective for understanding bone metabolic dysregulation under iron overload conditions.

## 1 Introduction

Osteoporosis is a systemic skeletal disorder characterized by reduced bone mass and microarchitectural deterioration, leading to a significantly elevated risk of fractures. Against the backdrop of accelerating global aging, it has emerged as a formidable public health challenge (Kerschan-Schindl, 2016; Khiyali et al., 2024). Epidemiological data indicate that the prevalence of osteoporosis among individuals aged 50 and above in China reaches 19.2%, with women being disproportionately affected (Morin et al., 2025). Bone homeostasis is maintained through a tightly regulated balance between osteoblast-mediated bone formation and osteoclast-mediated bone resorption, a process primarily governed by the RANKL/RANK/OPG signaling axis (Chi et al., 2023; Cavalcante et al., 2024). Currently, anti-resorptive agents, including bisphosphonates and RANKL monoclonal antibodies (e.g., denosumab), constitute the mainstay of clinical management (Kikuchi et al., 2023); however, their long-term administration has been associated with potential adverse effects such as osteonecrosis of the jaw and atypical fractures (Srivastava and Sapra, 2022; Cotts and Cifu, 2018; Song et al., 2022; Ghani and Arfee, 2023). Therefore, elucidating novel pathogenic mechanisms underlying osteoporosis and identifying safer and more effective therapeutic targets are of considerable clinical and scientific significance.

In recent years, the association between dysregulated iron metabolism and osteoporosis has attracted increasing attention. Iron overload, a common complication of hereditary hemochromatosis and thalassemia, has been established as an independent risk factor for osteoporosis (Sousa et al., 2020; Tatsumi et al., 2024; Sato et al., 2020). Clinical studies have demonstrated that bone mineral density is significantly reduced and fracture risk is markedly elevated in patients with iron overload (Barton et al., 2023; Pinto and Forni, 2020; Chan et al., 2023; Shah et al., 2004). Conventionally, excessive iron ions are thought to generate reactive oxygen species (ROS) *via* the Fenton reaction, triggering oxidative stress that directly impairs osteoblast function and promotes osteoclast differentiation (Tao et al., 2022; Zhou et al., 2024). However, this direct toxicity mechanism alone is insufficient to fully account for the complex pathological processes underlying iron overload-induced bone disease. Iron overload has been closely associated with ferroptosis, an iron-dependent form of programmed cell death that is characterized by the aberrant accumulation of lipid peroxides, and whose key regulatory mediators have been identified to include the pro-ferroptotic regulator acyl-CoA synthetase long-chain family member 4 (ACSL4) and the anti-ferroptotic regulator glutathione peroxidase 4 (GPX4). While the pathogenic involvement of ferroptosis has been well established across a wide spectrum of pathological conditions, its precise functional role in the pathogenesis of osteoporosis remains to be fully elucidated (Gao, 2022; Ma et al., 2022; Zhang et al., 2024). It has been demonstrated that iron chelators (e.g., deferoxamine, dfo) and ferroptosis inhibitors (e.g., ferrostatin-1, fer-1) can effectively mitigate iron overload-induced damage in various cell models, suggesting that ferroptosis represents a viable therapeutic target (Deng et al., 2021; Fang et al., 2020; Jiang Z. et al., 2022). Furthermore, as an important organelle for intracellular iron recycling and release, lysosome and its V-ATPase subunit *Atp6v0d2* have garnered growing interest for their roles in iron metabolism and cell death regulation. Nevertheless, the specific function of *Atp6v0d2* in iron overload-induced osteoporosis remains to be elucidated (Duan et al., 2018; Drakesmith et al., 2015; Rogers and Cahill, 2023; Stockwell et al., 2017).

The osteoimmune microenvironment plays a pivotal role in the regulation of bone metabolism, and the crosstalk between immune cells and bone cells constitutes a fundamental basis for the maintenance of bone homeostasis (Sapra et al., 2021). Studies have shown that immune cells can directly regulate bone remodeling through the secretion of various bioactive factors. For instance, neutrophils isolated from subjects with renal osteodystrophy have been demonstrated to indirectly promote osteoblastogenesis in bone marrow-derived macrophages, an effect that is mediated by modulation of the PI3K-Akt signaling pathway to drive cytokine release (Lu et al., 2026). In contrast, interferon-γ secreted by T cells has been reported to inhibit osteoclastogenesis *via* suppression of the RANKL-RANK signaling cascade (Takayanagi et al., 2000). Among these immune cell subsets, macrophages have emerged as central and non-redundant regulators of skeletal homeostasis, whose critical role in orchestrating bone remodeling processes has been widely recognized. Macrophages are characterized by remarkable phenotypic plasticity and can be polarized toward a pro-inflammatory M1 phenotype or a reparative M2 phenotype in response to distinct microenvironmental cues, participating in bone resorption and immune regulation through cytokine secretion and phagocytic activity (Xia et al., 2021; Yan et al., 2024; Yang et al., 2023). Notably, iron overload has been well documented to profoundly disrupt the osteoimmune microenvironment. It has been demonstrated by existing studies that iron overload can induce aberrant macrophage polarization, impair their immunomodulatory function, and disrupt the balance between bone formation and bone resorption, which in turn exacerbates the onset and progression of osteoporosis (Miron et al., 2023; Zhao et al., 2023; Hu et al., 2023; Song et al., 2023). Nevertheless, the precise mechanisms by which macrophages contribute to iron overload-associated osteoporosis remain to be fully elucidated.

Therefore, the study aimed to investigate the potential molecular mechanisms underlying iron overload-induced osteoporosis. First, an iron-overloaded rat model was established to evaluate the effects of iron overload on bone microarchitecture and cellular activity. Subsequently, MC3T3-E1 osteoblast precursor cells were employed together with transcriptomic analysis to explore the molecular mechanisms and key regulatory genes involved in iron overload-induced ferroptosis. Finally, conditioned medium derived from iron-overloaded macrophages was utilized to elucidate the mechanisms through which the pro-inflammatory immune microenvironment regulates osteogenic and osteoclastic functions. By integrating the dual perspectives of ferroptosis and the osteoimmune microenvironment, this study provides novel insights into the pathogenesis of iron overload-induced osteoporosis and offers a new perspective for understanding bone metabolism dysregulation under iron overload conditions.

## 2 Materials and methods

### 2.1 Animal model establishment and assessment

Male Sprague-Dawley rats aged 6–8 weeks with a body weight of (180 ± 20) g were obtained from the Animal Center of North Sichuan Medical College and housed in a specific pathogen-free (SPF) facility maintained at (25 ± 2) °C with *ad libitum* access to food and water. All animal experimental protocols were approved by the Experimental Animal Ethics Committee of North Sichuan Medical College (Approval No. NSMC-2024-036) and conducted strictly in accordance with the National Institutes of Health Guide for the Care and Use of Laboratory Animals. Rats were randomly assigned to a control group (NC group) and an iron overload model group (FAC group). Iron overload was induced by intraperitoneal injection of ferric ammonium citrate (FAC; 0.04 g/kg, dissolved in normal saline) three times per week for four consecutive weeks, while the NC group received an equivalent volume of normal saline. 72 h after the final administration, rats were anesthetized *via* intraperitoneal injection of pentobarbital sodium (30 mg/kg). Blood samples were collected *via* cardiac puncture. Subsequently, the rats were euthanized by cervical dislocation, and target tissues were dissected and isolated.

Femoral samples were harvested from experimental rats and fixed in 4% paraformaldehyde (Servicebio, Wuhan, China) at room temperature for 24 h, followed by decalcification in 0.5 M EDTA (Servicebio) for 4 weeks. The decalcified tissues were dehydrated through a graded ethanol series, embedded in paraffin, and sectioned at a thickness of 5 μm. H&E staining was conducted with a commercially available kit (Servicebio, Wuhan, China) following standard histological protocols to assess general bone tissue morphology. For evaluation of collagen fiber distribution and bone matrix morphology, Masson’s trichrome staining was performed using a dedicated staining kit (Servicebio, Wuhan, China) in accordance with the manufacturer’s instructions. Prussian blue staining was carried out using a Prussian blue iron staining kit (Solarbio, Beijing, China) to detect iron deposition in bone tissue. Tartrate-resistant acid phosphatase (TRAP) staining was performed using a TRAP staining kit (Sigma-Aldrich) in accordance with the manufacturer’s instructions, with TRAP-positive multinucleated cells identified as osteoclasts. All histological images were acquired using a digital slide scanner (Leica Aperio AT2, Wetzlar, Germany).

Bone microarchitecture was assessed by high-resolution micro-computed tomography (Micro-CT; Scanco Medical AG, Bassersdorf, Switzerland). Femora were fixed in 4% PFA for 24 h, rinsed three times with PBS, dehydrated in 75% ethanol, and placed in standardized cylindrical sample holders for scanning. The scanning parameters were set as follows: voltage, 70 kV; current, 200 μA; resolution, 18 μm. Three-dimensional reconstruction and quantitative analysis were performed on the trabecular bone region spanning slices 60 to 260 (200 slices in total) distal to the growth plate of the femur, as well as the cortical bone region comprising 100 consecutive slices beginning 100 slices proximal to the trabecular analysis region. Key bone morphometric parameters were quantified, including bone mineral density (BMD), bone volume fraction (BV/TV), trabecular number (Tb.N), trabecular separation (Tb.Sp), structure model index (SMI), and cortical thickness (Ct.Th).

Immunofluorescence was performed to evaluate the expression levels of target proteins in rat bone tissue, including ACSL4, osteopontin (OPN), forkhead box protein O1 (FOXO1), matrix metalloproteinase 9 (MMP9), GPX4, solute carrier family seven member 11 (SLC7A11), cluster of differentiation 86 (CD86), and arginase-1 (ARG-1). The distal femur samples were fixed, decalcified, and embedded in paraffin, then sectioned along the sagittal plane. Three serial sections per sample were used for quantitative analysis. All antibodies were obtained from Huaan, China. Tissue sections were deparaffinized, rehydrated, and subjected to antigen retrieval with citrate buffer (pH 6.0; MXB Biotechnologies, Fuzhou, China). Sections were then permeabilized, blocked, and incubated sequentially with primary antibodies and horseradish peroxidase (HRP)-conjugated secondary antibodies. Nuclei were counterstained with DAPI (Sigma-Aldrich). The trabecular bone region of the metaphysis, 0.5–1.0 mm below the growth plate, was selected as the field of view for fluorescence imaging, focusing on trabecular bone surfaces and surrounding cells within the bone marrow cavity. Images were acquired using a laser scanning confocal microscope (Olympus FV3000, Tokyo, Japan), and positively stained cells were quantified using ImageJ software.

Blood samples were collected *via* cardiac puncture and centrifuged at 3,500 rpm for 10 min to obtain serum. The following biomarkers were measured by enzyme-linked immunosorbent assay (ELISA): serum iron, alkaline phosphatase (ALP), osteocalcin (OC), vitamin D (VITD), interleukin-10 (IL-10), and tumor necrosis factor-α (TNF-α). Four weeks of iron overload intervention significantly altered the bone metabolic status of the model rats. Assays were performed following the manufacturer’s instructions. Briefly, standard, sample, and blank wells were prepared; samples and HRP-conjugated detection antibodies were added and incubated at 37 °C for 60 min. After thorough washing, substrate solution was added for chromogenic development, and absorbance was measured at 450 nm. Analyte concentrations were calculated from standard curves.

### 2.2 Cell culture and treatment

MC3T3-E1 were obtained from the Cell Bank of the Chinese Academy of Sciences and used as an osteoblast model. Cells were cultured in α-MEM medium (Gibco, USA) supplemented with 10% fetal bovine serum (FBS) and 100 IU/mL penicillin/streptomycin (both from Gibco, USA) at 37 °C in a 5% CO_2_ humidified incubator.

Mouse bone marrow-derived macrophages (BMDMs) were isolated from the femurs of 6–8-week-old Balb/c mice provided by the Animal Center of North Sichuan Medical College and served as an osteoclast model. After euthanasia and disinfection, the femurs and tibias were excised and immersed in PBS containing 10% penicillin/streptomycin for 5 min. Both ends of the bones were cut off, and the marrow cavity was flushed with complete medium. The collected bone marrow cells were seeded in culture dishes and incubated overnight. The supernatant was then collected, and red blood cells were lysed using red blood cell lysis buffer. The obtained BMDMs were resuspended in α-MEM medium and counted. Subsequently, the cells were cultured in α-MEM medium (Gibco, USA) containing 10% FBS and 100 IU/mL penicillin/streptomycin, with supplementation of 40 ng/mL M-CSF (Huabio, China) and 150 ng/mL RANKL (Huabio, China) every other day until mature osteoclasts were formed.

Mouse monocyte macrophage leukemia cells (RAW264.7) were purchased from the Cell Bank of the Chinese Academy of Sciences and used as a macrophage model. They were maintained in DMEM medium supplemented with 10% FBS and 100 IU/mL penicillin/streptomycin. All cells were cultured at 37 °C in a 5% CO_2_ atmosphere, and the medium was renewed every 3 days.

### 2.3 Cell viability and drug screening

To optimize the treatment conditions for FAC, cell viability was assessed using the CCK-8 assay. MC3T3-E1 cells were seeded into 96-well plates at a density of 5 × 10^3^ cells/well. After overnight incubation, the cells were treated with α-MEM medium containing 0, 10, 100, 300, or 500 μM FAC for 24, 48, and 72 h. At each time point, the medium was replaced with serum-free α-MEM containing 1% CCK-8 solution, and the plates were incubated at room temperature for 1 h. Absorbance was measured at 450 nm using a microplate reader. Cell viability (%) was calculated relative to the 0 μM FAC group to identify the optimal FAC concentration and intervention time. This condition was designated as the FAC group, and the control group was denoted as the NC group. All experiments were performed with at least three replicates.

To determine the optimal concentrations of ferroptosis inhibitors, MC3T3-E1 cells were seeded in 96-well plates (5 × 10^3^ cells/well). After overnight culture, the cells were pretreated with α-MEM containing 1, 5, or 10 μM fer-1 (MCE, America) or dfo (MCE, America) for 8 h, followed by incubation with α-MEM containing 300 μM FAC for an additional 48 h. Based on the results, cells pretreated with 5 μM fer-1 were assigned to the fer-1 group and those pretreated with 5 μM dfo were assigned to the dfo group. To further verify the specificity of ferroptosis in iron overload-induced osteoblast injury, cells were pretreated with the pan-caspase apoptosis inhibitor Z-VAD-FMK (ZV; MCE, America) at 1.875, 3.75, or 7.5 μM for 8 h, followed by 300 μM FAC treatment for 48 h. In parallel, cells were pretreated with the autophagic flux inhibitor hydroxychloroquine (HCQ; MCE, America) at 0.625, 1.25, or 2.5 μM for 8 h, followed by the same FAC treatment. Cell viability in all experiments was assessed by the CCK-8 assay.

To evaluate the effect of FAC on RAW264.7 cell proliferation, RAW264.7 cells were seeded in 96-well plates at 5 × 10^3^ cells/well. After overnight incubation, the medium was replaced with fresh DMEM containing 0, 50, 100, 300, or 500 μM FAC. On days 1, 2, and three of culture, cell viability was assessed using the CCK-8 assay. The absorbance values were normalized to that of the 0 μM FAC group on day 1, and cell viability (%) was calculated for each treatment group.

To investigate the effect of macrophage-conditioned medium on MC3T3-E1 cells, RAW264.7 cells were seeded in 6-well plates at 1 × 10^6^ cells/well and treated with 200 μM FAC for 48 h. The supernatant was collected by centrifugation and mixed with α-MEM medium at volume ratios of 1:2, 1:1, and 2:1 to prepare inflammatory conditioned medium (ICM). MC3T3-E1 cells were then cultured with these conditioned media, and cell viability was measured by CCK-8 assay on days 1, 2, and 3. Negative control (plain α-MEM, NC group) and positive control (α-MEM directly supplemented with 200 μM FAC, FAC-200 group) were included. Cell viability (%) was calculated relative to the NC group on day 1. The mixture at a volume ratio of 1:1 (raw ICM: α-MEM) with 48 h incubation, which reduced MC3T3-E1 viability to approximately 70% of the NC group, was selected as the standard ICM treatment condition for all subsequent experiments.

To screen the optimal conditions for IL-17 pathway antagonist treatment, MC3T3-E1 cells were seeded in 96-well plates at 5 × 10^3^ cells/well. After 24 h of culture, the medium was discarded. Negative control (blank medium), positive control (ICM), and experimental groups were established. IL-17A antagonist 1 (MCE, America, Cat. No. HY-101913) was used as the inhibitor of the IL-17 pathway and is hereafter referred to as IL-17AI. IL-17AI is a small-molecule antagonist that specifically binds the central dimer interface pocket of the IL-17A homodimer, thereby disrupting the IL-17A/IL-17 receptor A (IL-17 R A) interaction. In the experimental groups, cells were pretreated with various concentrations of IL-17AI for either 1 h or 6 h, after which the medium was replaced with ICM and cultured for an additional 48 h. The concentrations used for the 1 h pretreatment were 82.7 nM, 165.5 nM, 331 nM, and 331 μM, while those for the 6 h pretreatment were 82.7 nM, 165.5 nM, and 331 nM. Cell viability (%) was calculated relative to the negative control group to determine the optimal concentration and treatment duration of the IL-17 pathway antagonist.

### 2.4 Osteoblast differentiation and functional assays

After drug treatment, MC3T3-E1 cells were cultured in osteogenic differentiation medium for 14 days, with the medium replaced every 3 days. The osteogenic differentiation medium consisted of α-MEM supplemented with 10% fetal bovine serum (FBS), 10 mM β-glycerophosphate, 50 μg/mL L-ascorbic acid-2-phosphate, 10 nM dexamethasone, and 1% penicillin/streptomycin, all purchased from Sigma-Aldrich (China). Following culture, ALP staining and activity quantification, alizarin red staining, quantitative real-time PCR (RT-qPCR), and Western blot analyses were performed.

For ALP staining, cells were fixed with 4% paraformaldehyde and incubated with BCIP/NBT staining solution in the dark for 30 min, then observed under a light microscope (Nikon Eclipse E100, Nikon, Japan). For quantitative ALP activity, cells were lysed with RIPA buffer, and the lysate was centrifuged at 3,000 *g* for 20 min. The supernatant was incubated with chromogenic substrate at room temperature for 30 min, and absorbance was measured at 405 nm. Total protein concentration in the lysate was determined using a BCA assay, and ALP activity was normalized to total protein.

For alizarin red staining, fixed cells were stained with alizarin red solution (Solarbio, China) for 10 min at room temperature, rinsed with PBS, and observed for mineralized nodule formation under a microscope.

RT-qPCR was performed to assess mRNA expression levels. Total RNA was extracted using an RNA extraction kit (Vazyme, Shanghai, China), and its concentration and purity were measured with a microspectrophotometer, with A260/A280 ratios between 1.8 and 2.0. One microgram of total RNA was reverse transcribed into cDNA using PrimeScript RT Master Mix (Vazyme) under the following conditions: 37 °C for 15 min and 85 °C for 5 s. RT-qPCR was conducted using SYBR Green Master Mix (Vazyme) on a CFX96 system (Bio-Rad, USA). Each 20 μL reaction contained 1 μL of cDNA and 0.4 μM each of forward and reverse primers. The thermal cycling conditions were 95 °C for 30 s, followed by 40 cycles of 95 °C for 5 s and 60 °C for 30 s. All reactions were performed in triplicate, and melting curve analysis was used to verify primer specificity. Relative gene expression was calculated using the 2^−ΔΔCt method with Gapdh as the internal control. Primer sequences are listed in Supplementary Table S1.

Western blot analysis was conducted to detect protein expression. After drug treatment, cells were lysed in RIPA buffer (Yamei, China) on ice for 30 min and centrifuged at 12,000 *g*. Total protein concentration was determined using a BCA assay (Solarbio, China). Equal amounts of protein were separated by SDS-PAGE (Bio-Rad, USA) and transferred onto PVDF membranes (Bio-Rad, USA). Membranes were blocked with 5% non-fat milk at 4 °C for 1 h, followed by incubation with primary antibodies overnight at 4 °C. After washing with TBST, membranes were incubated with HRP-conjugated secondary antibodies (1:10,000) at room temperature for 1 h. Protein bands were visualized using an enhanced chemiluminescence (ECL) system and imaged with a ChemiDoc XRS system (Bio-Rad, Germany). Densitometric analysis was performed using ImageJ software, and relative protein expression was normalized to the internal control β-actin. Antibody information is provided in Supplementary Table S2.

### 2.5 Cell cycle analysis

Cell cycle distribution was analyzed using a cell cycle assay kit. MC3T3-E1 cells were seeded into culture plates at a density of 1 × 10^5^ cells/well and treated according to the experimental protocol. Cells were collected, adjusted to a concentration of 1 × 10^6^/mL, and fixed with pre-chilled 70% ethanol overnight at 4 °C. Staining was performed following the manufacturer’s instructions, and samples were analyzed using a flow cytometer with red fluorescence detection at 488 nm excitation wavelength to determine the proportion of cells in each phase of the cell cycle.

### 2.6 Intracellular reactive oxygen species measurement

Intracellular ROS levels were assessed using the DCFH-DA fluorescent probe. MC3T3-E1 cells were seeded into 24-well plates at a density of 1 × 10^4^ cells/well and treated according to the experimental groups. After treatment, the medium was discarded, and cells were incubated with fresh medium containing 10 μM DCFH-DA at 37 °C in the dark for 20 min. Cells were gently washed twice with PBS and counterstained with Hoechst 33,342 (5 μg/mL) for 10 min. Fluorescence images were captured using a fluorescence microscope. For quantitative analysis, fluorescence intensity was measured at excitation wavelength 488 nm and emission wavelength 525 nm using a microplate reader to evaluate intracellular ROS levels.

### 2.7 Ferroptosis-related biochemical assays

MC3T3-E1 cells were seeded into 6-well plates at a density of 1 × 10^5^ cells/well and treated according to the experimental design. Cells were collected and lysed with RIPA buffer on ice for 30 min, followed by centrifugation at 10,000×g for 10 min at 4 °C. The supernatant was collected for subsequent measurements of Fe^2+^ concentration, MDA content, and reduced GSH levels.

Intracellular Fe^2+^ concentration was determined using a commercial assay kit. The supernatant was incubated with Fe^2+^ detection working solution at room temperature for 10 min, and absorbance was measured at 593 nm. Fe^2+^ concentration was calculated based on a standard curve.

MDA content was assessed using an MDA assay kit. The supernatant was mixed with MDA detection working solution and heated in a boiling water bath for 15 min. Two hundred microliters of the supernatant were transferred to a 96-well plate, and absorbance was measured at 532 nm. MDA content was calculated using a standard curve.

GSH levels were analyzed using a GSH assay kit. The supernatant was incubated with GSH chromogenic reagent for 5 min, and absorbance was measured at 405 nm. Intracellular GSH content was determined using a standard curve.

### 2.8 Mitochondrial function assays

Mitochondrial morphology was examined by transmission electron microscopy. MC3T3-E1 cells were seeded into 6-well plates at a density of 1 × 10^5^ cells/well. After overnight culture and treatment, cells were collected, fixed with glutaraldehyde and osmium tetroxide, dehydrated through a graded ethanol series, and embedded in epoxy resin. Ultrathin sections were double-stained with lead citrate and uranyl acetate and observed under a transmission electron microscope for mitochondrial ultrastructure.

Western blot analysis was performed to detect the protein expression levels of key ferroptosis regulatory proteins, including GPX4, SLC7A11, and transferrin receptor 1 (TFRC). Detailed experimental procedures are described in Section 2.4.

### 2.9 Osteoclast differentiation and functional assays

BMDMs were seeded into 24-well plates and cultured overnight in α-MEM containing 40 ng/mL M-CSF. The medium was then replaced with α-MEM containing 40 ng/mL M-CSF and 150 ng/mL RANKL to induce osteoclast differentiation. The medium was refreshed every 2 days until mature osteoclasts were formed.

Osteoclast differentiation was evaluated by TRAP staining. Cells were fixed with methanol and incubated with TRAP staining working solution at room temperature for 1 h. TRAP-positive cells appeared purple-red under light microscopy, and TRAP activity was assessed based on the morphology, staining intensity, and number of positive cells.

Bone resorption activity was assessed using a bone slice resorption assay. Sterilized bone slices (Yangming Biotechnology, China) were placed in 96-well plates, seeded with BMDMs, and induced to differentiate into osteoclasts as described above. When obvious resorption pits were observed under microscopy, cells were removed, and bone slices were fixed, dehydrated, dried, and examined under a scanning electron microscope. The area and number of resorption pits per bone slice were quantified as indicators of osteoclast activity and bone resorption capacity.

Western blot analysis was performed to detect the protein expression levels of key osteoclast functional and regulatory proteins: matrix metalloproteinase 9 (MMP9), cathepsin K (CTSK), nuclear factor kappa B p65 subunit (P65), phosphorylated nuclear factor kappa B p65 subunit (p-P65), inhibitor of nuclear factor kappa B alpha (IκBα), and phosphorylated inhibitor of nuclear factor kappa B alpha (p-IκBα). Detailed experimental procedures are described in Section 2.4.

### 2.10 Macrophage phenotype and inflammatory response assessment

Flow cytometry was performed to assess macrophage polarization by detecting surface markers. RAW264.7 cells were seeded in 6-well plates and treated with 0, 50, or 200 μM FAC for 48 h. After collection, cells were incubated with anti-CD16/CD32 antibody to block non-specific binding, followed by staining with PE-conjugated anti-CD86 antibody and Alexa Fluor 647-conjugated anti-CD206 antibody in the dark. Stained cells were resuspended in staining buffer and analyzed using a flow cytometer, with 2 × 10^4^ events collected per sample. Data were analyzed using FlowJo™ v10.8.1 software, and the CD86^+^/CD206^+^ ratio was used to evaluate M1/M2 polarization tendency.

Inflammatory cytokine levels in macrophage culture supernatants were measured by ELISA, and the expression of inflammation-related genes was detected by RT-qPCR to assess the effects of iron overload on macrophage inflammatory responses.

### 2.11 Transcriptome sequencing and bioinformatics analysis

MC3T3-E1 cells in the logarithmic growth phase were evenly seeded in culture plates and treated according to the experimental protocol, with three biological replicates per group. After treatment, total RNA was extracted using an RNA extraction kit and stored at −80 °C until further use. Transcriptome sequencing was performed by OE Biotech Co., Ltd. Sequencing libraries were constructed and sequenced on the Illumina Novaseq 6,000 platform. After quality control of raw data, clean reads were aligned to the reference genome using HISAT2. Gene expression levels were quantified using HTSeq-count and normalized to fragments per kilobase of transcript per million mapped reads (FPKM). Sample correlation analysis was performed to assess the consistency of gene expression profiles, with Pearson correlation coefficients |r| > 0.9 indicating good reproducibility. Differential expression analysis was conducted using DESeq2, with significantly differentially expressed genes (DEGs) defined as |log2 fold change| > 1 and *p <* 0.05. Hierarchical clustering analysis was performed using R software (v 3.2.0) to visualize gene expression patterns across samples. Volcano plots were generated to display the overall distribution of upregulated and downregulated DEGs. Gene Ontology and KEGG pathway enrichment analyses were performed to elucidate the biological functions and signaling pathways associated with the DEGs. Representative DEGs enriched in downregulated pathways were compared and presented as a heatmap. Gene set enrichment analysis (GSEA) was further performed to evaluate the enrichment of ferroptosis- and inflammation-related gene sets between groups, with normalized enrichment score (NES) and nominal p-value used to determine significant enrichment.

### 2.12 Plasmid construction and validation

Short hairpin RNA (shRNA) plasmids targeting *Atp6v0d2* and a non-targeting negative control shRNA plasmid (sh-NC) were constructed by APExBIO (Shanghai, China) using the pLent-U6-shRNA-CMV-Puro vector.

For MC3T3-E1 cells, 1 day before transfection, cells were seeded in antibiotic-free medium to reach 70%–80% confluence. For each well of a 24-well plate, 0.5 μg of shRNA plasmid DNA was diluted in 25 μL of serum-free medium followed by the addition of 1 μL of Lipo3K-A Reagent (APExBIO, China). In parallel, 1.5 μL of Lipo3K-B Reagent was diluted in 25 μL of serum-free medium and incubated at room temperature for 5 min. The DNA complex and Lipo3K-B dilution were combined, gently mixed, and incubated for 20 min at room temperature to form DNA–liposome complexes, which were then added to the cells. 6 h after transfection, the medium was replaced with fresh culture medium, and the cells were treated with FAC at the indicated concentrations for 48 h before subsequent analyses.

For BMDM-derived osteoclasts, BMDMs were first cultured with 40 ng/mL M-CSF for 2 days to allow adherence and macrophage differentiation. On day 2, transfection was performed using the same shRNA plasmids and Lipo3K transfection protocol described above. After transfection 6 h, the medium was replaced with fresh medium containing 40 ng/mL M-CSF, and FAC was added at the indicated concentrations for 48 h. Thereafter, the medium was replaced with differentiation medium containing 40 ng/mL M-CSF and 150 ng/mL RANKL, and the cells were continuously cultured until osteoclast differentiation was complete for downstream analyses.

Knockdown efficiency was validated at the mRNA levels by RT-qPCR. Cells transfected with sh-NC served as controls. Only groups with significant knockdown efficiency were used for subsequent experiments.

### 2.13 Statistical analysis

All experiments were performed with at least three independent biological replicates, each conducted in technical triplicate. Data are expressed as mean ± standard deviation (SD). Group differences were analyzed by one-way analysis of variance (ANOVA), followed by Tukey’s multiple pairwise comparison test for all intergroup comparisons (GraphPad Prism 9.0). A p-value <0.05 was considered statistically significant.

## 3 Results

### 3.1 Iron overload inhibits osteoblast function and promotes osteoclast function *in vivo*

Micro-CT three-dimensional reconstruction and quantitative analysis revealed that iron overload induced significant structural damage to Male Sprague-Dawley rats bone. As shown in Figures 1A,B, BV/TV was significantly decreased in the FAC group compared with the NC group (*p <* 0.05). Meanwhile, the FAC group exhibited reduced Tb.N and increased Tb.Sp, alongside a significantly elevated SMI (*p <* 0.05), indicating a transition from plate-like to rod-like trabecular morphology. Cortical bone analysis showed a decreasing trend in Ct.Th in the FAC group.

**FIGURE 1:**
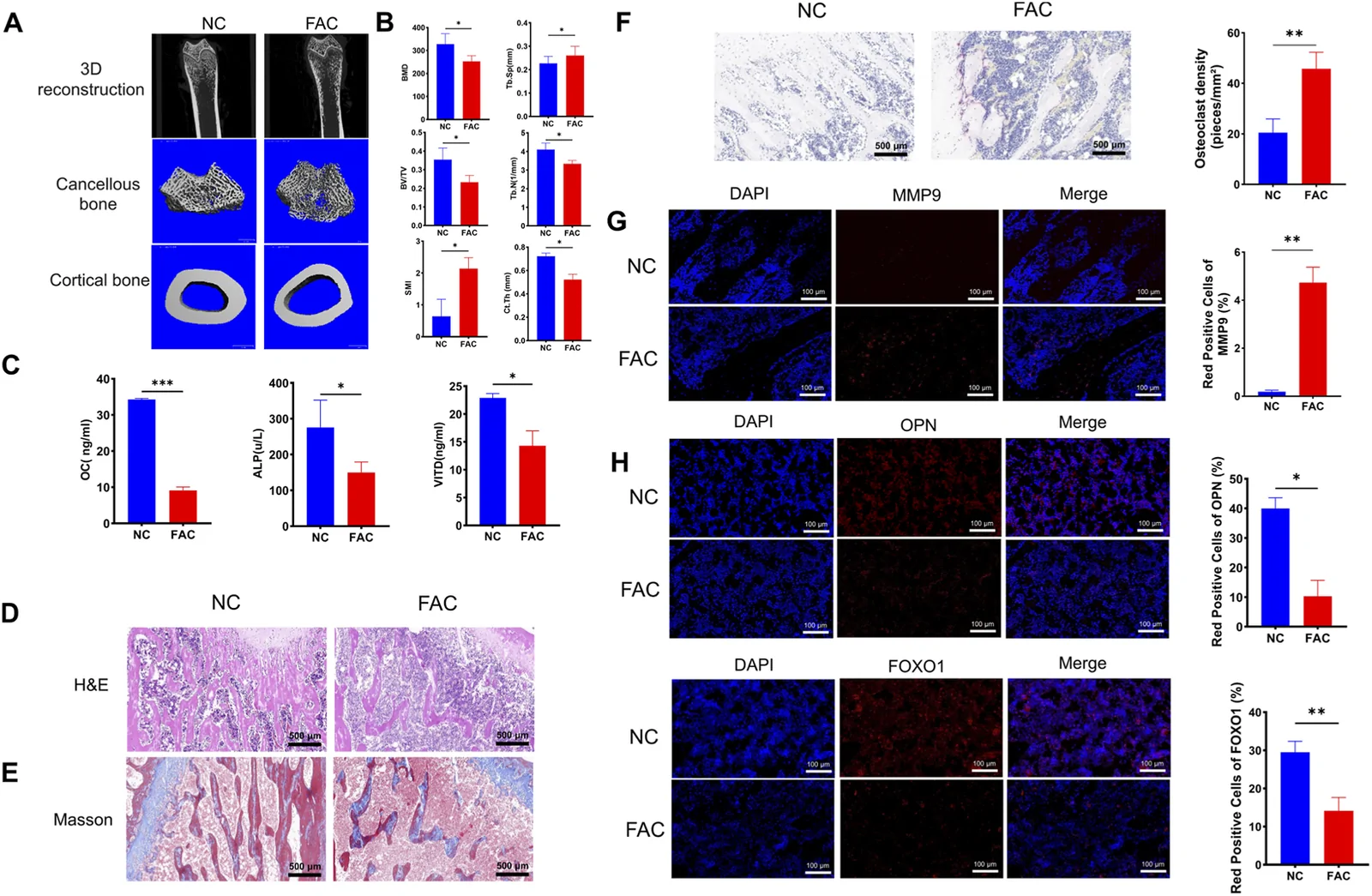
Iron overload impairs bone metabolism and distal femoral metaphyseal microarchitecture in Sprague‐Dawley rats **(A)** Micro-CT images of the distal femoral metaphysis **(B)** Quantitative micro-CT analysis of bone parameters **(C)** ELISA results showed serum levels of osteogenic markers OC, ALP, and VITD **(D,E)** H&E and Masson staining of bone sections (10×, scale bar = 500 μm) **(F)** TRAP staining and quantification of osteoclast density (10×, scale bar = 500 μm) **(G)** Immunofluorescence staining of MMP9 (20×, scale bar = 100 μm) **(H)** Immunofluorescence staining of OPN and FOXO1 (20×, scale bar = 100 μm). *p < 0.05, **p < 0.01, ***p < 0.001, n = 5. Unlabeled comparisons were not statistically significant.

Four weeks of iron overload intervention significantly altered the bone metabolic status of the model rats. Serum biochemical analysis demonstrated that the levels of OC, ALP, and VITD were significantly decreased in the FAC group compared with the NC group (Figure 1C).

Histomorphological results further confirmed these changes. H&E staining (Figure 1D) showed sparse trabeculae with enlarged inter-trabecular spaces in the proximal femoral growth plate region of the FAC group, consistent with the Micro-CT findings. Masson staining (Figure 1E) revealed reduced red-stained areas representing mature bone matrix and loosely structured blue-stained areas representing newly formed bone and collagen in the FAC group, suggesting disrupted bone turnover balance and potentially enhanced bone resorption.

To evaluate the direct effects of iron overload on bone cell activity, functional markers of osteoclasts and osteoblasts were examined. TRAP is a specific marker enzyme for osteoclasts. TRAP staining (Figure 1F) showed that both the number of osteoclasts and the average cell area were increased in the FAC group compared with the NC group, suggesting that iron overload may promote osteoclast differentiation. MMP9 is a marker protein for osteoclasts. Immunofluorescence staining (Figure 1G) revealed that the MMP9 fluorescence positivity rate was significantly lower in the NC group than in the FAC group (*p <* 0.01), indicating that iron overload promotes osteoclast activity.

Regarding osteoblasts, immunofluorescence staining was performed to detect osteogenesis-related protein expression. OPN is a key component of the bone matrix and plays an indispensable role in bone formation and development. FOXO1 is widely recognized to be involved in body development, aging, cell viability, and oxidative stress in various organs and cells; FOXO1 also regulates amino acid uptake, thereby modulating protein synthesis in osteoblasts. As shown in Figure 1H, the fluorescence signal intensities of OPN and FOXO1 in bone tissue were significantly weakened and the number of positive cells was reduced in the FAC group compared with the NC group, indicating that iron overload inhibited the expression of these two key osteogenesis-related factors.

Collectively, these results demonstrate that iron overload leads to decreased serum osteogenic markers, degradation of bone microarchitecture, trabecular sparseness, and loosely structured bone matrix. These findings are consistent with the quantified changes in bone mineral density and microstructural parameters, collectively reflecting the disruptive effects of iron overload on bone homeostasis in rats.

### 3.2 Iron overload promotes ferroptosis and exacerbates inflammation in bone tissue

To validate the establishment of the iron overload model and examine iron distribution *in vivo*, tissue iron deposition and serum iron levels were analyzed. Prussian blue staining revealed abundant blue iron particle deposits in the liver and femoral tissues of FAC-treated rats, whereas minimal deposits were observed in the NC group (Figure 2A). Further analysis demonstrated that serum iron levels were significantly higher in the FAC group compared with the NC group. These results confirm that intraperitoneal FAC injection successfully established a systemic iron overload model, with excessive iron accumulation in the liver and bone tissue.

**FIGURE 2:**
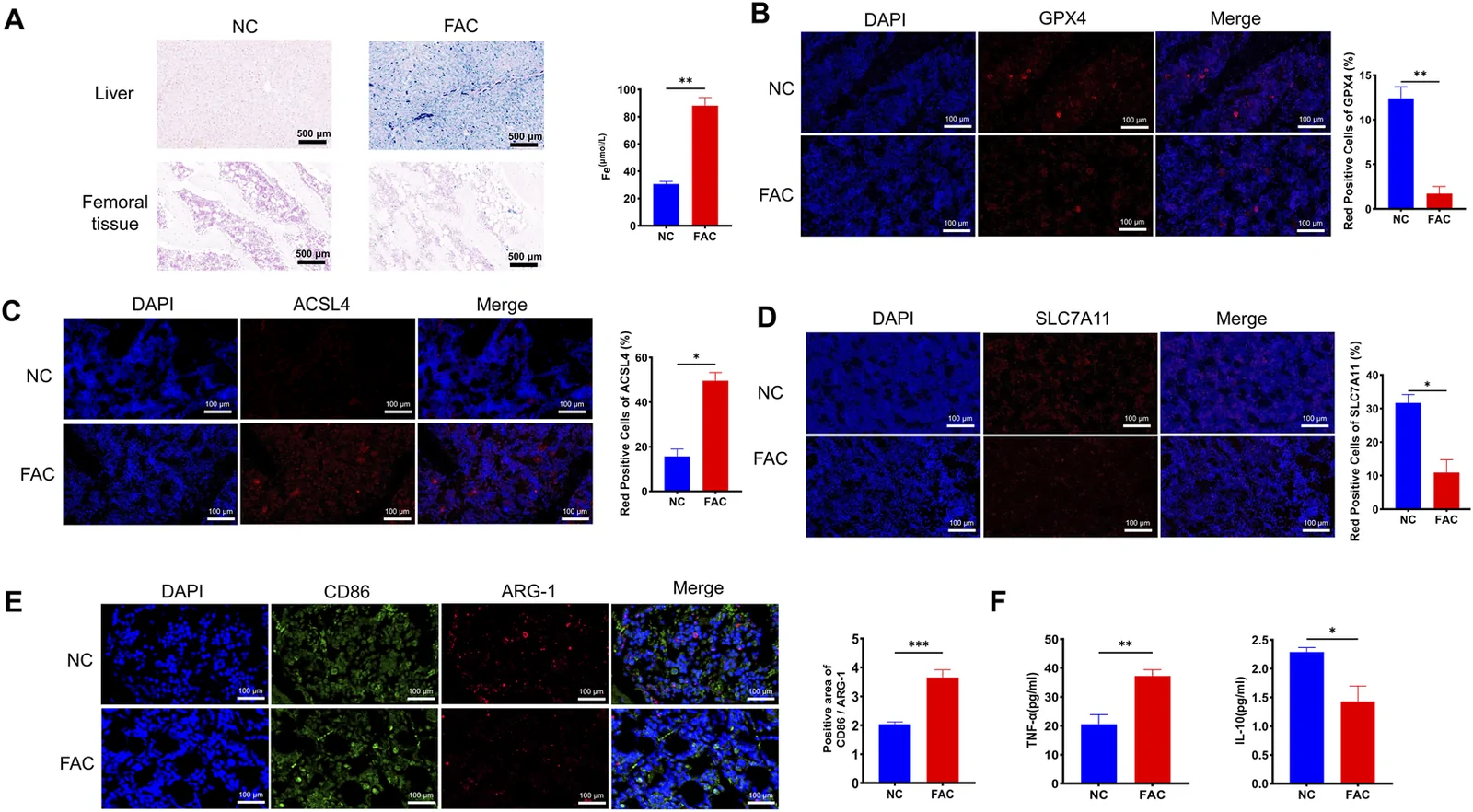
Iron overload induces ferroptosis and inflammatory response in rats **(A)** Prussian blue staining of liver and femoral tissue (10×, scale bar = 500 μm) **(B–D)** Immunofluorescence staining and quantification of GPX4, ACSL4 and SLC7A11 in distal femoral metaphysis (20×, scale bar = 100 μm) **(E)** Immunofluorescence staining and quantification of CD86 and ARG-1 in distal femoral metaphysis (20×, scale bar = 100 μm) **(F)** Serum levels of TNF-α and IL-10 measured by ELISA. *p < 0.05, **p < 0.01, ***p < 0.001, n = 5. Unlabeled comparisons were not statistically significant.

The reaction between ferrous ions and hydrogen peroxide generates highly reactive hydroxyl radicals, which induce lipid peroxidation and ferroptosis through oxidative damage—a process known as the Fenton reaction. To investigate the pathogenic mechanisms of iron overload in bone tissue, the expression of key ferroptosis-regulating proteins was examined. Immunofluorescence staining showed that the fluorescence positivity rate of the pro-ferroptosis protein ACSL4 was significantly increased in the femoral tissue of the FAC group compared with the NC group, while the positivity rates of the anti-ferroptosis proteins GPX4 and SLC7A11 were significantly decreased (Figures 2B–D). These findings indicate that iron overload disrupts the regulatory balance of ferroptosis in bone tissue, potentially driving lipid peroxidation and ferroptosis through ACSL4 upregulation and GPX4/SLC7A11 downregulation. The increased ACSL4 expression and decreased GPX4 and SLC7A11 expression collectively suggest enhanced lipid peroxidation and impaired ferroptosis defense mechanisms. As an iron-dependent form of cell death, ferroptosis may directly contribute to reduced osteoblast number and function, providing a mechanistic explanation for the previously observed decreases in osteogenic markers, impaired bone formation, and downregulation of osteogenic proteins such as OPN and FOXO1.

The immune system is closely associated with bone metabolism, with differentially polarized macrophages secreting functionally distinct cytokines. M1 macrophages release pro-inflammatory cytokines including TNF-α and IL-6, which promote bone resorption. Serum cytokine levels were measured and immunofluorescence staining of femoral tissue was performed. The results demonstrated that the mean fluorescence intensity of the M1 macrophage marker CD86 was significantly higher in the FAC group than in the NC group, while expression of the M2 marker ARG-1 was markedly decreased, resulting in a substantially increased CD86/ARG-1 ratio (Figure 2E). Serum cytokine analysis revealed significantly elevated concentrations of the pro-inflammatory cytokine TNF-α and reduced levels of the anti-inflammatory cytokine IL-10 in the FAC group (Figure 2F). These findings indicate that iron overload not only drives local macrophage polarization toward the pro-inflammatory M1 phenotype in bone tissue but also induces a systemic pro-inflammatory state. The imbalanced M1/M2 macrophage ratio in bone tissue and altered systemic pro-inflammatory/anti-inflammatory cytokine profiles suggest that iron overload elicits a significant inflammatory response. M1 macrophage polarization and increased pro-inflammatory cytokines such as TNF-α may directly inhibit osteoblast activity while promoting osteoclast differentiation and exacerbating bone resorption, consistent with the previously observed trabecular structural deterioration and bone loss. Notably, a potential interplay exists between inflammation and ferroptosis: inflammatory cytokines can exacerbate oxidative stress, while ferroptosis-released contents may further amplify inflammatory signals, creating a vicious cycle that collectively drives bone metabolic imbalance.

In summary, these *in vivo* findings suggest that iron overload may induce osteoporosis through dual mechanisms: directly promoting osteoblast ferroptosis and impairing bone formation, while driving macrophage M1 polarization and chronic inflammation that disrupts bone remodeling balance. Both pathways may contribute to the pathogenesis of iron overload-induced osteoporosis. Based on these observations, subsequent investigations examined the effects of iron overload on osteogenic cells and macrophages *in vitro*.

### 3.3 Iron overload induces ferroptosis in MC3T3-E1 cells and impairs osteogenic differentiation

To investigate the cytotoxic effects of iron overload on MC3T3-E1 cells and identify potential intervention strategies, concentration screening was first performed. Following treatment with various concentrations of FAC for 24 h, cell viability was significantly reduced in the 300 μM and 500 μM groups. After 48 h of treatment, significant inhibition of cell viability was observed at concentrations of 100 μM and above. By 72 h, cell viability was significantly decreased in all treatment groups (Figure 3A). Since treatment with 300 μM FAC for 48 h reduced cell viability to approximately 70% of the control level, this condition was selected to establish an *in vitro* iron overload model for subsequent experiments.

**FIGURE 3:**
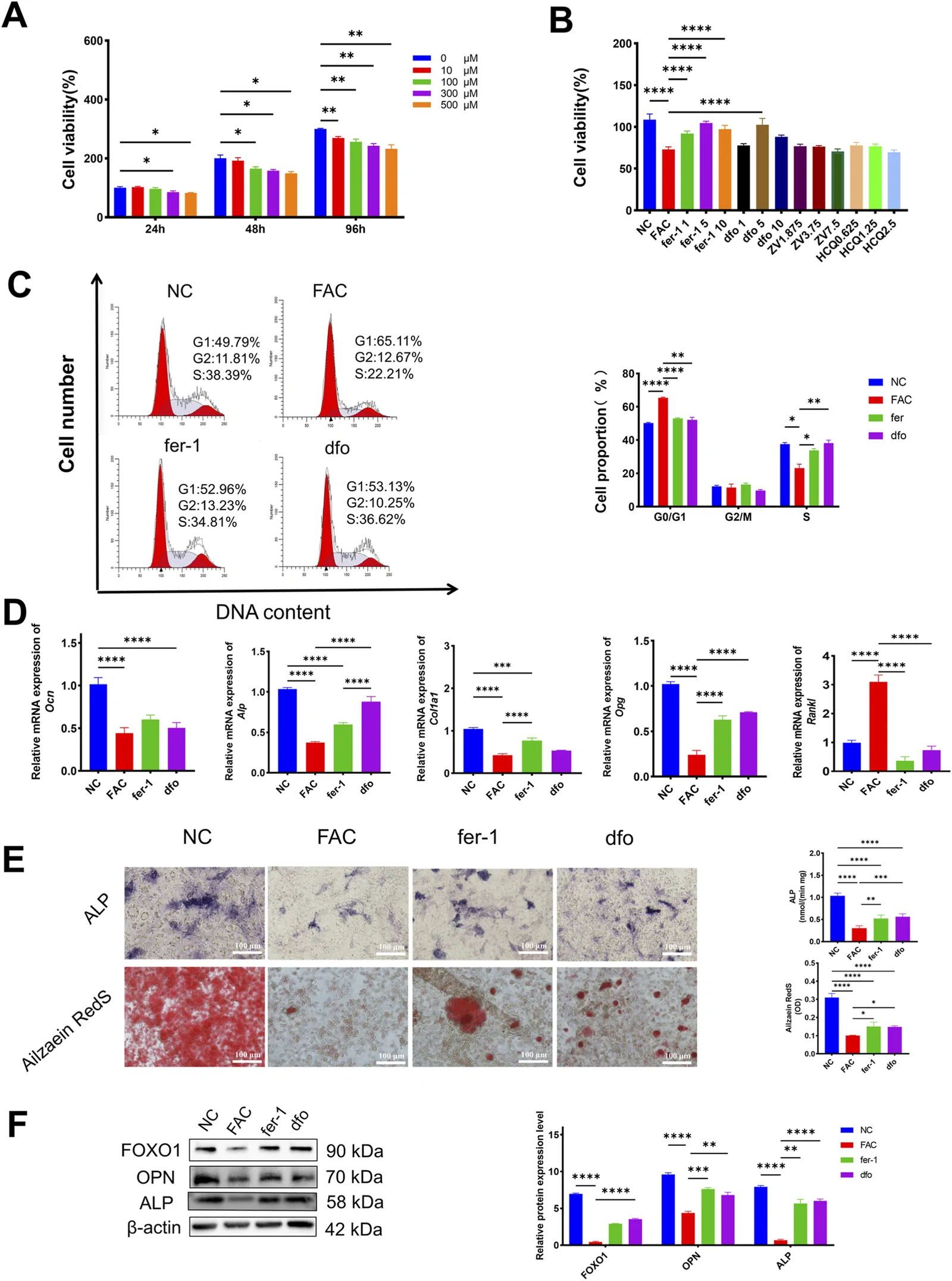
Iron overload inhibits MC3T3-E1 cell viability and impairs osteogenic differentiation, which are reversed by ferroptosis inhibitors **(A)** CCK-8 assay of cell viability **(B)** CCK-8 assay of cell viability following treatment with fer-1 and dfo **(C)** Flow cytometric analysis of cell cycle distribution **(D)** RT-qPCR analysis of OCN, ALP, COL1A1, OPG, and RANKL mRNA expression **(E)** ALP and Alizarin Red S staining for ALP activity and mineralized nodule formation (10×, scale bar = 200 μm) **(F)** Western blot analysis of FOXO1, OPN, and ALP protein expression. *p < 0.05, **p < 0.01, ***p < 0.001, ****p < 0.0001, n = 3. Unlabeled comparisons were not statistically significant.

To explore effective protective measures, the effects of different inhibitors were examined. Cells were pretreated with fer-1 or dfo for 8 h, followed by exposure to 300 μM FAC for 48 h. The results showed that pretreatment with 5 μM fer-1 or 5 μM dfo significantly restored cell viability, whereas no additional protective effect was observed at 10 μM compared with 5 μM. In contrast, the apoptosis inhibitor Z-VAD or the autophagy inhibitor hydroxychloroquine slightly increased viability, but the differences were not statistically significant (Figure 3B). Therefore, 5 μM was selected as the optimal concentration for both fer-1 and dfo in subsequent experiments.

Cell cycle distribution was further analyzed by flow cytometry (Xiao et al., 2025). Compared with the control group, FAC treatment resulted in a significantly increased proportion of cells in the G0/G1 phase and a significantly decreased proportion in the S phase. Notably, pretreatment with fer-1 or dfo effectively reversed these changes (Figure 3C). These results indicate that iron overload induces G0/G1 phase arrest in MC3T3-E1 cells, which can be alleviated by inhibiting ferroptosis or chelating iron ions.

Osteogenic differentiation was assessed through multiple approaches. RT-qPCR analysis demonstrated that FAC treatment significantly downregulated the mRNA expression of key osteogenic markers, including *Ocn*, *Alp* and *Col1a1* in MC3T3-E1 cells compared with the control group. Additionally, iron overload disrupted the regulatory balance of osteoclast differentiation, as evidenced by significantly decreased mRNA expression of *Opg* and markedly upregulated expression of *Rankl*. These gene expression alterations were effectively reversed by fer-1 or dfo treatment (Figure 3D). ALP staining after 7 days of osteogenic induction revealed significantly reduced ALP activity in the FAC group, indicating impaired early osteogenic differentiation; this reduction was markedly reversed by fer-1 and dfo treatment (Figure 3E). Alizarin red S staining showed that the number and area of mineralized nodules were markedly decreased in the FAC group, suggesting severely compromised late-stage mineralization capacity, whereas fer-1 or dfo intervention significantly promoted nodule formation. The expression levels of osteogenesis-related proteins OPN, FOXO1, and ALP were significantly decreased in the FAC group compared with the control group, while fer-1 or dfo treatment partially restored their expression (Figure 3F).

Ferroptosis is a form of programmed cell death mediated by the accumulation of lipid peroxides, and its regulatory mechanism is distinct from those of apoptosis and necrosis. The core features of ferroptosis include glutathione depletion and reduced activity of GPX4, which prevent the normal metabolism and clearance of lipid peroxides. Lipid oxidation is then catalyzed by ferrous ions to generate ROS, ultimately triggering ferroptosis (Xia et al., 2021). Intracellular iron accumulation is a hallmark of ferroptosis, and increased ferrous ion levels can specifically enhance oxidative stress. Ferric ions are reduced to ferrous ions and stored in the labile iron pool. Due to their high reducibility and reactivity, ferrous ions interact with unsaturated fatty acids *via* the Fenton reaction, inducing massive ROS production and promoting ferroptosis.

The results of the present study demonstrated that iron overload induced significant intracellular ferrous ion accumulation, which was effectively attenuated by treatment with fer-1 and dfo. The underlying mechanism is that dfo chelates intracellular free iron ions, and fer-1 scavenges free iron by forming complexes (Jiang H. et al., 2022). Iron overload exposes cells to oxidative stress. As reported by Karim et al. (Karim et al., 2022), in chondrocytes treated with high concentrations of FAC, iron overload not only disrupts cellular iron homeostasis but also induces oxidative stress and impairs normal cellular function. In this study, the effects of iron overload on lipid peroxidation and iron homeostasis were assessed using biochemical indicators. Colorimetric assays showed that compared with the control group, the FAC-treated group exhibited significantly increased MDA content (*p <* 0.0001), markedly decreased GSH levels (*p <* 0.0001), and substantially elevated intracellular Fe^2+^ levels (*p <* 0.0001) (Figure 4A). Treatment with fer-1 and dfo significantly reduced MDA and Fe^2+^ levels and partially restored GSH content. At the molecular level, RT-qPCR and Western blot analyses revealed (Figures 4B,C) that FAC treatment significantly downregulated the mRNA and protein expression of GPX4 and SLC7A11, which are negative regulators of ferroptosis, while significantly upregulating the expression of TFRC, a key protein responsible for iron uptake. These iron overload induced molecular abnormalities were reversed to varying degrees by fer-1 and dfo treatment.

**FIGURE 4:**
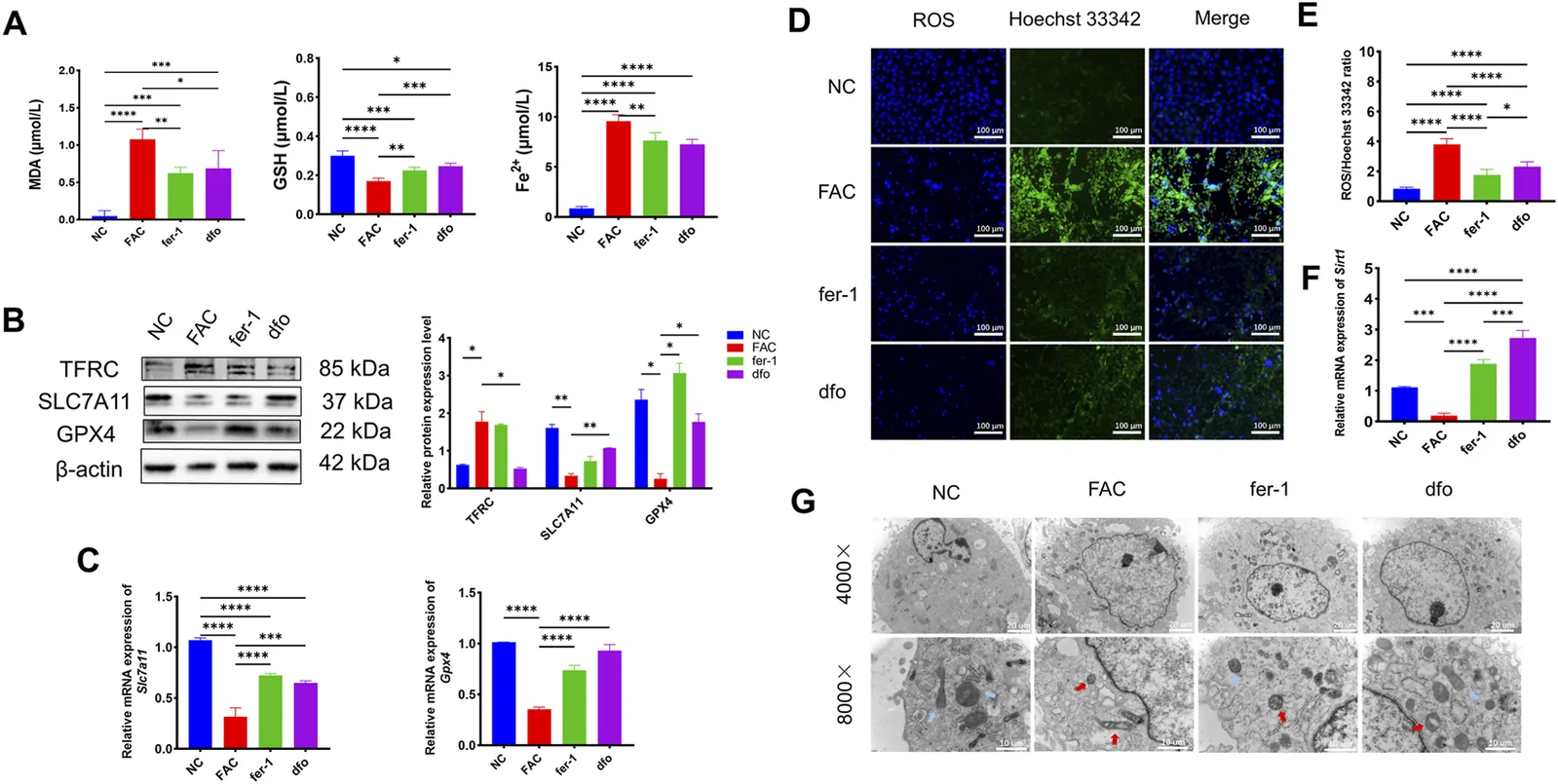
Iron overload induces ferroptosis, oxidative stress, and mitochondrial damage in MC3T3-E1 cells, which are reversed by ferroptosis inhibitors **(A)** Biochemical analysis of MDA, GSH, and Fe^2+^ levels **(B)** Western blot analysis of TFRC, SLC7A11, and GPX4 protein expression **(C)** RT-qPCR analysis of *Slc7a11* and *gpx4* mRNA expression **(D–E)** ROS fluorescence staining and quantification (20×, scale bar = 100 μm) **(F)** RT-qPCR analysis of ferroptosis-related gene expression **(G)** Transmission electron microscopy of mitochondrial ultrastructure (4,000×, scale bar = 20 μm; 8,000×, scale bar = 10 μm). *p < 0.05, **p < 0.01, ***p < 0.001, ****p < 0.0001, n = 3. Unlabeled comparisons were not statistically significant.

There is a close association between oxidative stress and ferroptosis. Jiang et al. (Jiang et al., 2026) found that icariside II induces apoptosis and ferroptosis in non-small cell lung cancer cells by impairing cellular antioxidant capacity (inhibiting Nrf2 activity), promoting the accumulation of lipid peroxidation substrates (ACSL4 activation), and triggering oxidative stress. In the present study, intracellular oxidative stress levels were detected, and DCFH-DA fluorescent probe assay results (Figures 4D,E) showed that compared with the control group, the intracellular ROS levels in the FAC-treated group increased sharply (*p <* 0.0001), while pretreatment with fer-1 or dfo significantly inhibited the excessive production of ROS (*p <* 0.0001). RT-qPCR analysis (Figure 4F) revealed that the mRNA expression level of Sirt1, a core antioxidant regulatory factor, was significantly downregulated in the FAC group (*p <* 0.0001), and this downregulation could be partially reversed by fer-1 and dfo intervention.

Studies have shown that mitochondria exhibit obvious morphological changes during ferroptosis. Tian et al. (Tian et al., 2016)reported that high iron concentrations increase mitochondrial permeability, leading to cytochrome C-mediated activation of Caspase-3, accompanied by upregulated Bax expression and downregulated Bcl-2 expression, thereby inducing mitochondrial apoptosis and osteoblast apoptosis. In contrast, N-acetylcysteine can reverse iron-induced mitochondrial dysfunction, effectively inhibit ROS production, and block cell apoptosis. Analysis of mitochondrial ultrastructure and function in this study showed that iron overload caused typical ferroptosis-related damage in MC3T3-E1 cells. Transmission electron microscopy observations (Figure 4G) demonstrated that mitochondria in the control group had a normal morphology with clear membrane structures and regular cristae, whereas mitochondria in the FAC-treated group were significantly atrophied with increased membrane density and significantly reduced cristae. These results confirm that iron overload disrupts cellular iron metabolic homeostasis: intracellular Fe^2+^ accumulation and upregulated TFRC expression provide substrates for the Fenton reaction, while downregulated GPX4 and SLC7A11 expression impairs one of the most important antioxidant defense systems of cells, preventing effective clearance of lipid peroxides and ultimately triggering ferroptosis. Specifically, fer-1 intervenes in this process by enhancing antioxidant defense, and dfo by reducing iron availability, both reversing the abnormal expression of key proteins, which further confirms that this pathway is the core mechanism underlying iron overload-induced death of osteogenesis-related cells.

### 3.4 Iron overload may promote osteoporosis by upregulating Atp6v0d2 expression

To investigate the mechanistic basis of iron overload-induced bone disorders, transcriptome sequencing was performed on MC3T3-E1 cells treated with or without FAC. All samples exhibited high sequencing quality (Q30 > 94.8%, total genome mapping rate > 96.7%), and principal component analysis and Pearson correlation analyses confirmed clear intergroup separation and good intragroup reproducibility (Supplementary Tables S1–S3; Supplementary Figure S3). Differential expression analysis revealed that iron overload resulted in 873 downregulated and 724 upregulated genes (Figure 5A). GSEA further confirmed significant enrichment of ferroptosis-related pathways in the FAC group (Figure 5B), consistent with the hypothesis that iron overload drives ferroptosis in preosteoblastic cells. GO and KEGG enrichment analyses showed that upregulated genes were primarily enriched in cell cycle and Rap1 signaling pathways related to cell proliferation, whereas downregulated genes were significantly enriched in biological processes such as osteoblast differentiation and bone mineralization, as well as signaling pathways closely associated with osteogenesis including TNF, Calcium, MAPK, and Wnt pathways (Figures 5C–F).

**FIGURE 5:**
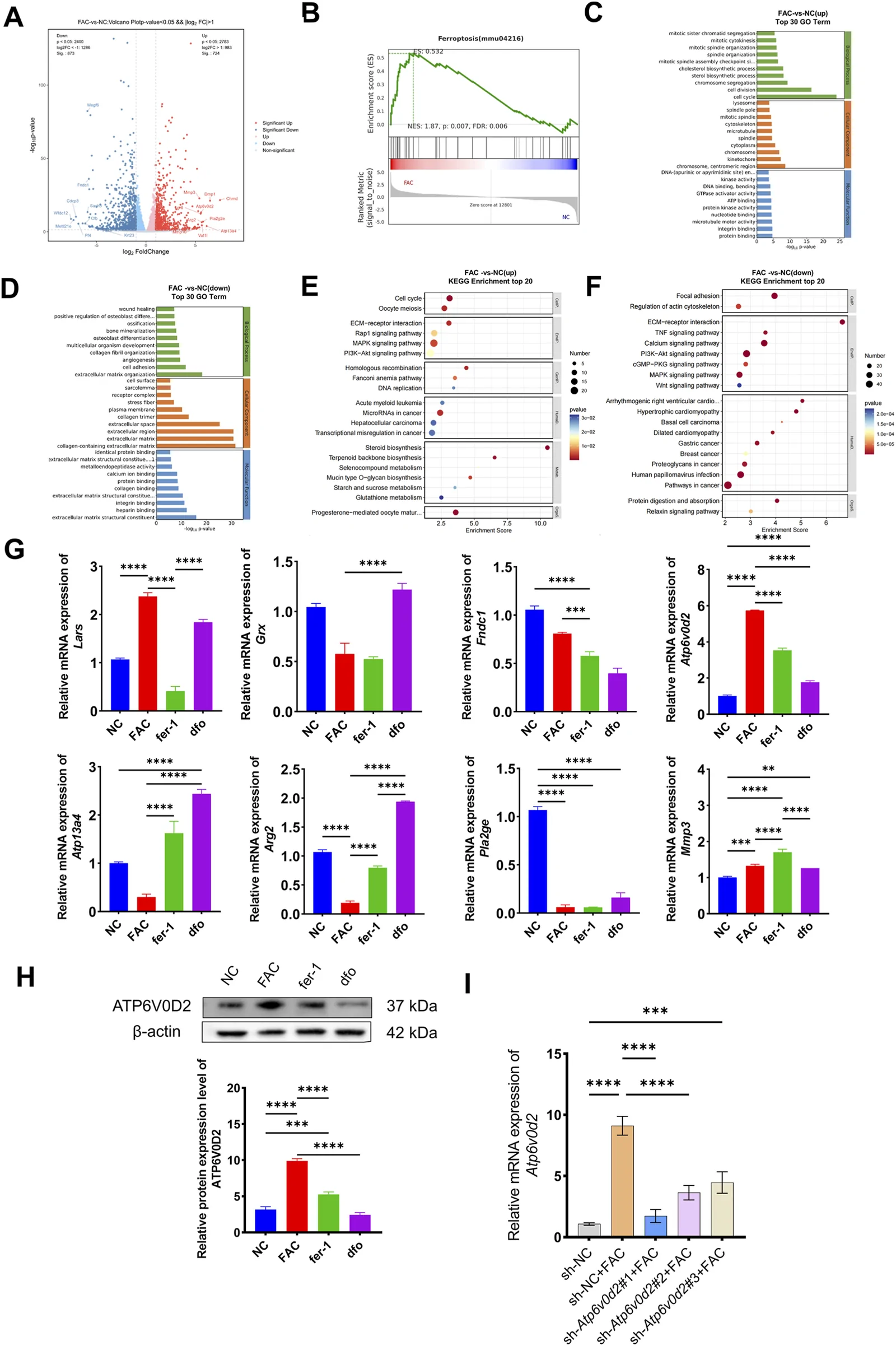
Iron overload induces differential gene expression in MC3T3-E1 cells and identifies *Atp6v0d2* as a key regulatory gene **(A)** Volcano plot of differential gene expression **(B)** GSEA enrichment analysis of ferroptosis-related pathways **(C–F)** KEGG pathway and GO functional enrichment analyses **(G)** RT-qPCR analysis of *Atp6v0d2* and other gene expression **(H)** Western blot analysis of ATP6V0D2 protein expression **(I)** RT-qPCR validation of Atp6v0d2 knockdown efficiency in MC3T3-E1 cells. *p < 0.05, ***p < 0.001, ****p < 0.0001, n = 3. Unlabeled comparisons were not statistically significant.

Based on transcriptome data from the NC and FAC groups, eight candidate genes were selected from the differentially expressed genes for RT-qPCR validation, of which five (Atp6v0d2, Fndc1, Lars, Grx, Mmp3) showed NC/FAC expression trends consistent with the RNA‐seq data (Figure 5G). To precisely identify core genes specifically involved in the iron overload–ferroptosis axis, we employed a stringent four group validation strategy (NC, FAC, FAC + fer‐1, FAC + dfo) by RT‐qPCR. Candidate genes were retained only if they met two criteria: (i) concordant NC/FAC expression trends with RNA‐seq, and (ii) simultaneous reversal of FAC‐induced alterations by both fer-1 and dfo. Among the five concordant candidates, only Atp6v0d2 satisfied both requirements—its FAC‐induced upregulation was reversed by both fer‐1 and dfo, while the other four were reversed by only one intervention or neither, and were thus excluded from further analysis. Atp6v0d2 encodes a core V‐ATPase subunit that regulates lysosomal acidification and intracellular iron homeostasis, serving as a key upstream ferroptosis regulator and participating in bone metabolism (Qi et al., 2020; Wu et al., 2009). Combined with validation results and functional relevance, it was identified as the core gene for subsequent mechanistic studies. Further Western blot analysis confirmed that FAC significantly upregulated ATP6V0D2 protein expression, which was reversed by fer-1 and dfo pretreatment, consistent with mRNA findings and confirming specific regulation by iron overload at both transcriptional and translational levels (Figure 5H).

To explore the functional role of *Atp6v0d2*, *Atp6v0d2* knockdown cell lines were established in MC3T3-E1 cells. RT-qPCR validation demonstrated that transfection with shRNA plasmids (*Atp6v0d2*#1 and *Atp6v0d2*#2) significantly reduced *Atp6v0d2* mRNA levels compared with the negative control (Figure 5I), and these two constructs were selected for subsequent experiments.

To evaluate the effect of *Atp6v0d2* knockdown on ferroptosis, oxidative stress and lipid peroxidation markers were assessed. DCFH-DA fluorescence staining revealed significantly elevated intracellular ROS levels in the sh-NC + FAC group under iron overload conditions, which were markedly attenuated in *Atp6v0d2* knockdown groups (*Atp6v0d2*#1/2 + FAC) (Figure 6A). Quantitative analysis showed that *Atp6v0d2* knockdown reduced the iron overload-induced ROS/Hoechst ratio by 56% and 53%, respectively (*p <* 0.0001, Figure 6B). Meanwhile, MDA content, a lipid peroxidation end product, was significantly increased in the sh-NC + FAC group compared with the control group, whereas MDA levels were decreased by 45% and 40% in *Atp6v0d2* knockdown groups (*p <* 0.01, Figure 6C). These results demonstrate that *Atp6v0d2* knockdown attenuates iron overload-induced oxidative stress injury by suppressing ROS generation and lipid peroxidation.

**FIGURE 6:**
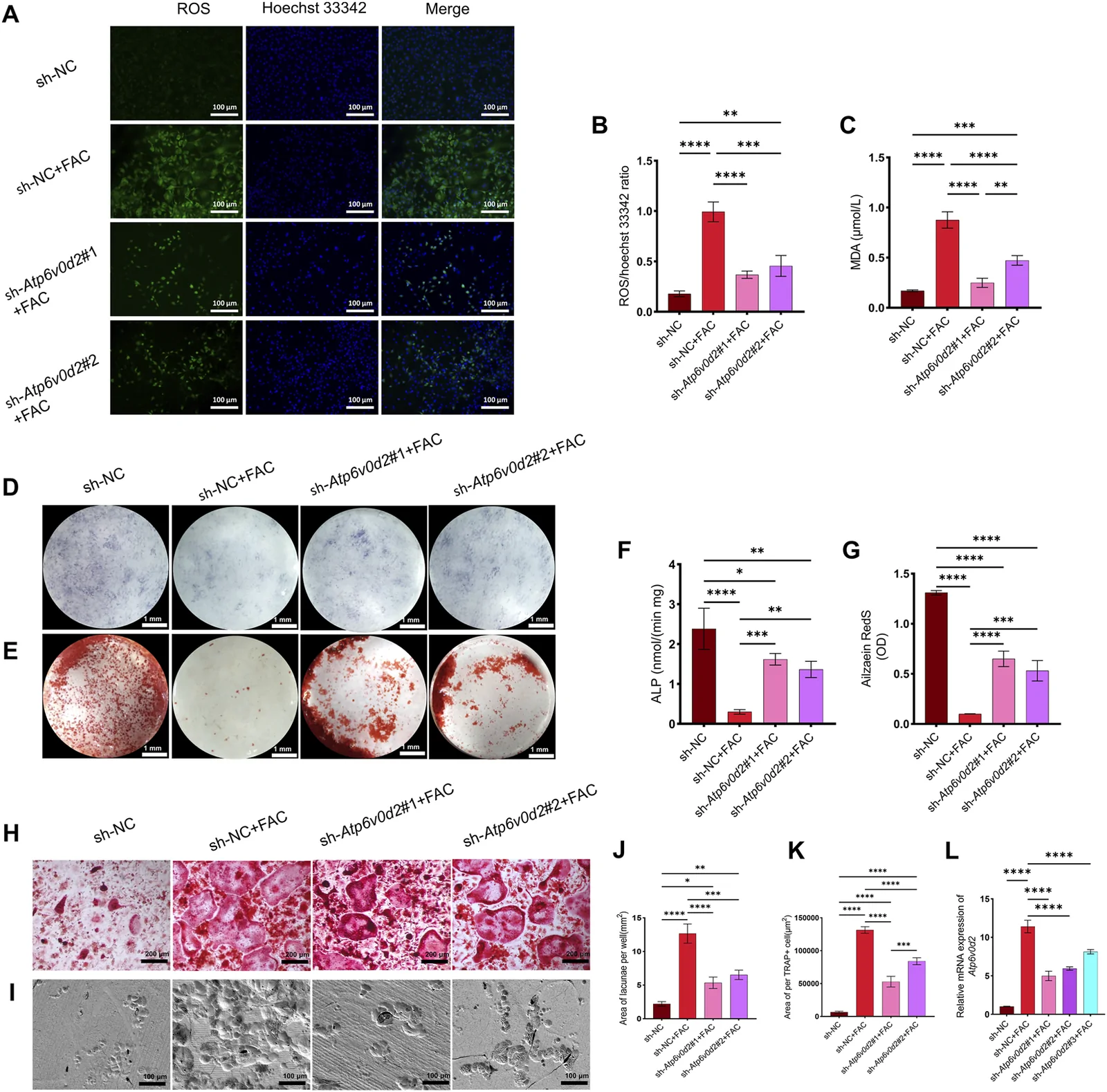
*Atp6v0d2* knockdown alleviates osteoblast dysfunction and inhibits osteoclast hyperactivation under iron overload **(A,B)** ROS fluorescence staining and quantification (20×, scale bar = 100 μm) **(C)** MDA content assay **(D,F)** ALP staining (1×, scale bar = 1 mm) and quantification **(E,G)** Alizarin Red S staining (1×, scale bar = 1 mm) and quantification **(H,J)** TRAP staining (10×, scale bar = 200 μm) and quantification of osteoclast area **(I,K)** Scanning electron microscopy (20×, scale bar = 100 μm) and quantification of bone resorption pit area **(L)** RT-qPCR validation of Atp6v0d2 knockdown efficiency in BMDMs. *p < 0.05, **p < 0.01, ***p < 0.001, ****p < 0.0001, n = 3. Unlabeled comparisons were not statistically significant.

To investigate the regulatory role of *Atp6v0d2* knockdown on osteogenic and osteoclastic functions, functional validation assays were performed. ALP staining and activity quantification after 7 days of osteogenic induction revealed significantly restored ALP activity in *Atp6v0d2* knockdown groups (#1 and #2) compared with the sh-NC + FAC group (Figures 6D,F). Alizarin red S staining after 14 days of osteogenic induction showed that *Atp6v0d2* knockdown significantly promoted mineralized nodule formation (Figures 6E,G).

The efficiency of Atp6v0d2 knockdown in BMDMs was confirmed by RT-qPCR, which showed that Atp6v0d2 mRNA levels were significantly reduced in both sh-Atp6v0d2#1 and sh-Atp6v0d2#2 groups compared with the sh-NC control (Figure 6L). Regarding osteoclast differentiation, *Atp6v0d2* knockdown significantly suppressed iron overload-induced osteoclast hyperactivation. TRAP staining revealed that the number, size, and TRAP-positive area of osteoclasts were markedly increased in the sh-NC + FAC group compared with the control group, whereas these parameters were significantly reduced in *Atp6v0d2* knockdown groups (sh-*Atp6v0d2*#1 + FAC and sh-*Atp6v0d2*#2 + FAC) relative to the sh-NC + FAC group (Figures 6H,J). Bone slice resorption assays further confirmed that iron overload enhanced the bone-degrading capacity of osteoclasts, while *Atp6v0d2* knockdown significantly reduced the resorption pit area and resulted in shallower pit morphology, indicating suppressed bone resorption activity (Figures 6I,K).

### 3.5 Iron overload drives macrophage polarization toward a pro-inflammatory phenotype

Macrophages, derived from the monocyte lineage, function as immune sentinels and maintain homeostasis by recognizing and eliminating pathogens, adapting to microenvironmental changes through phenotypic polarization (Yan et al., 2024). To investigate the effects of iron overload on macrophages, RAW264.7 cells were treated with various concentrations of FAC. CCK-8 assay revealed a dose-and time-dependent decrease in cell viability (Figure 7A). After 48 h of treatment, cell viability in the 50 μM and 200 μM FAC groups was reduced to 78% and 61% of the control level, respectively. Based on these results, 50 μM and 200 μM FAC for 48 h were selected as low-and high-concentration treatment conditions for subsequent experiments.

**FIGURE 7:**
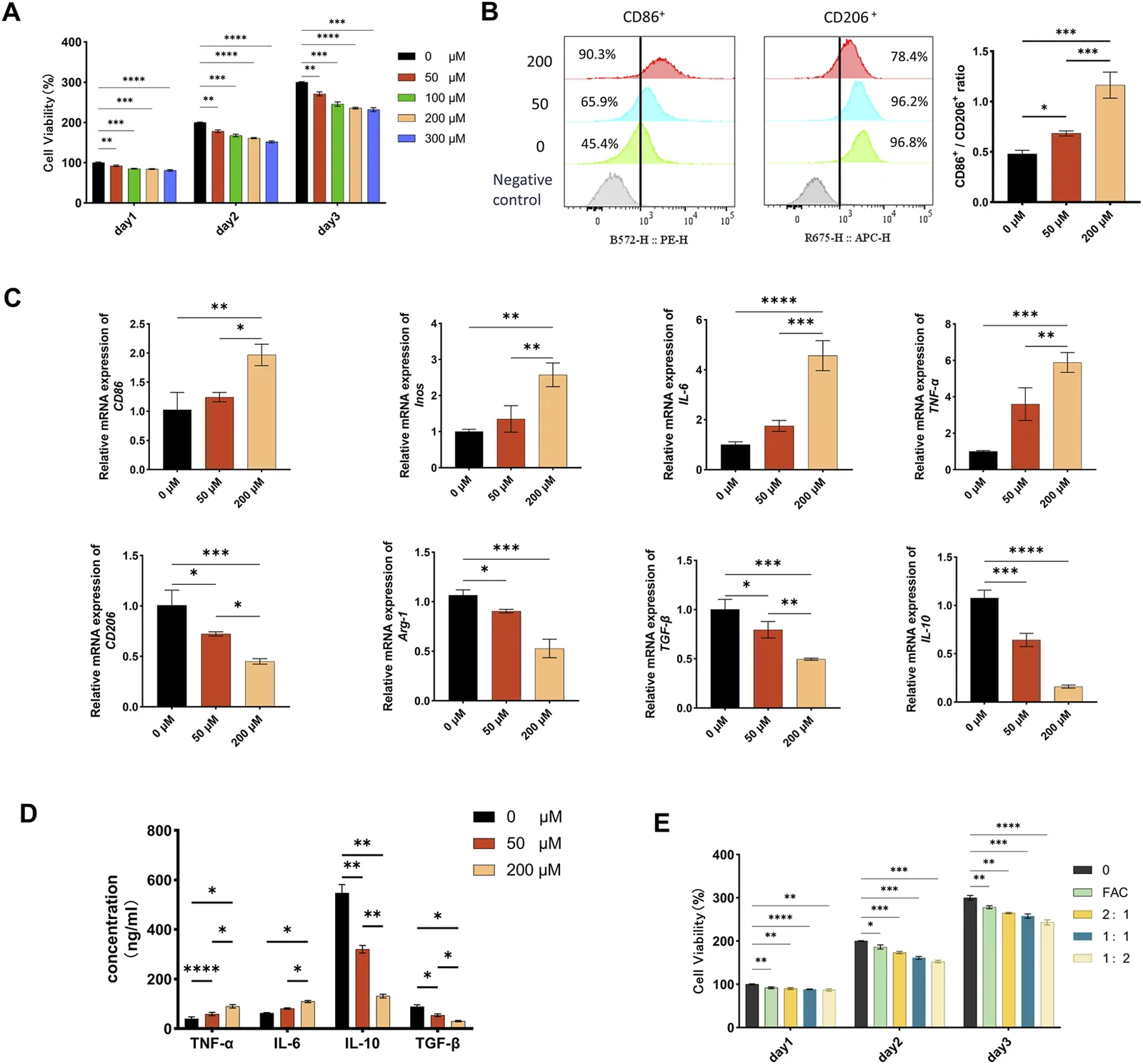
Iron overload inhibits macrophage viability and drives M1 pro-inflammatory polarization **(A)** CCK-8 assay of RAW264.7 cell viability **(B)** Flow cytometric analysis of CD86 and CD206 expression in macrophages **(C)** RT-qPCR analysis of CD86, TNF, Il-6, CD206, Il-10, and TGF-β mRNA expression in macrophages **(D)** ELISA of TNF-α, IL-6, IL-10, and TGF-β levels in macrophage culture supernatants **(E)** CCK-8 assay of MC3T3-E1 cell viability using conditioned media from FAC-treated macrophages. *p < 0.05, **p < 0.01, ***p < 0.001, ****p < 0.0001, n = 3. Unlabeled comparisons were not statistically significant.

To evaluate the effect of iron overload on macrophage polarization, flow cytometry was performed to detect the expression of the M1 marker CD86 and the M2 marker CD206. The results showed that treatment with 50 μM FAC significantly increased the CD86^+^/CD206^+^ ratio compared with the control group, while 200 μM FAC further elevated this ratio (Figure 7B), indicating that iron overload drives macrophage polarization toward the pro-inflammatory M1 phenotype in a dose-dependent manner. These findings are consistent with previous reports that iron promotes M1 polarization through the NF-κB pathway (Wu et al., 2009; Guggisberg et al., 2022; Guggisberg and Ryu, 2019; Jiang et al., 2024; Jähn et al., 2017; Yang et al., 2019). At the transcriptional level, RT-qPCR analysis revealed that both 50 μM and 200 μM FAC treatments significantly upregulated the mRNA expression of M1 markers (*CD86*, *Inos, IL-6*, *TNF-*α) and downregulated the expression of M2 markers (*CD206*, *Arg-1*, *TGF-β*, *IL-10*), with more pronounced changes observed in the high-concentration group (Figure 7C). At the protein secretion level, ELISA further confirmed that FAC treatment promoted the secretion of pro-inflammatory cytokines TNF-α and IL-6 in a concentration-dependent manner, while inhibiting the release of anti-inflammatory cytokines *IL-10* and *TGF-β* (Figure 7D). These results demonstrated that iron overload not only regulates the transcription of polarization-related genes in macrophages but also directly affects their inflammatory secretory profile, collectively driving the microenvironment toward a pro-inflammatory state. To investigate the effects of macrophage-derived factors on MC3T3-E1 under iron overload conditions, conditioned medium was collected from RAW264.7 macrophages treated with 200 μM FAC for 48 h MC3T3-E1 cells were then exposed to various dilutions of this conditioned medium. As shown in Figure 7E, cell viability decreased in a time- and conditioned-medium-concentration-dependent manner. After 48 h of treatment, mixed media prepared at fresh complete medium to conditioned medium supernatant ratios of 2:1, 1:1, and 1:2 (corresponding to low, medium, and high concentrations of conditioned medium, respectively) reduced cell viability to 73.5%, 61.0%, and 52.5% of control levels, respectively, compared with 86.3% in the direct 200 μM FAC treatment group (FAC-200). Based on these results, the 1:1 ratio of fresh complete medium to conditioned medium supernatant for 48 h was selected as the inflammatory conditioned medium (ICM) treatment for subsequent experiments.

### 3.6 Iron overload macrophage-derived ICM disrupts osteoblast homeostasis

To explore the impact of iron overload macrophage-derived inflammatory conditioned medium on osteoblast homeostasis and to exclude the direct cytotoxic effect of residual iron in ICM, five experimental groups were set in this assay: blank control group (NC, cultured with plain α-MEM), direct iron overload group (FAC-200, cultured with fresh α-MEM supplemented with 200 μM FAC, serving as the control for direct free iron stimulation), ICM treatment group, ICM combined with ferroptosis inhibitor Fer-1 group (ICM + fer-1), and ICM combined with iron chelator DFO group (ICM + dfo).

The intracellular ROS levels were assessed by DCFH-DA staining (Figures 8A,B). Strong green fluorescence was observed in the FAC-200 group, indicating marked ROS production, while the ICM group exhibited weaker fluorescence. Quantitative analysis using a fluorescence microplate reader revealed that the ROS/Hoechst 33,342 fluorescence intensity ratio was significantly elevated in both the FAC-200 and ICM groups compared with the control group, confirming that both treatments induced oxidative stress. However, no significant differences in ROS levels were observed between the ICM group and the ICM + fer-1 or ICM + dfo groups. The lower ROS signal in the ICM group can be explained by the detection principle of DCFH-DA that the probe can only detect intracellular ROS in living cells, and membrane rupture of dead cells leads to ROS release, which cannot be effectively captured by this method.

**FIGURE 8:**
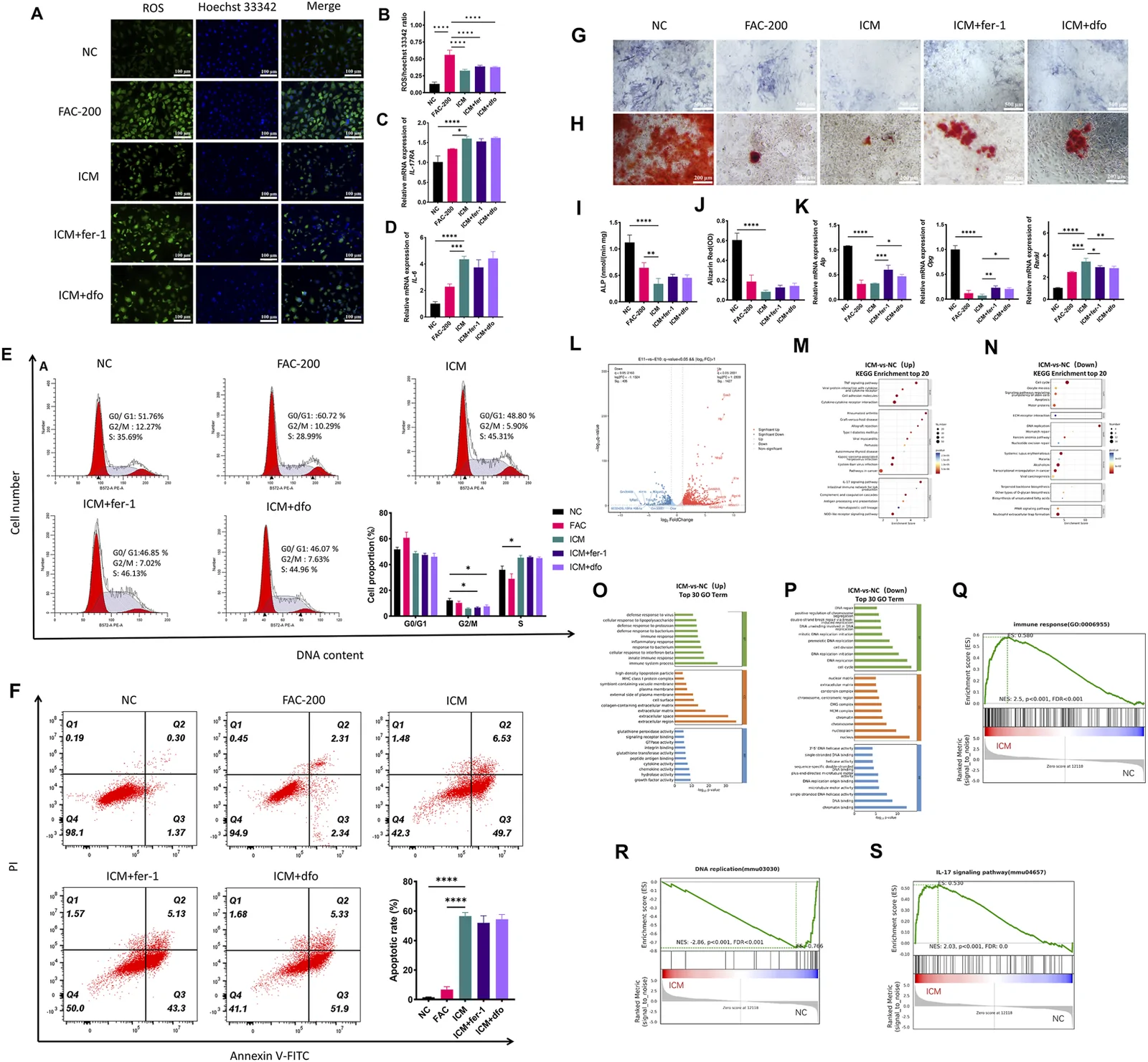
ICM impairs MC3T3-E1 cell function, and ferroptosis inhibitors exert a partial rescue effect **(A,B)** ROS fluorescence staining (20×, scale bar = 100 μm) and quantification **(C,D)** RT-qPCR analysis of proinflammatory factor mRNA expression **(E)** Flow cytometric analysis of cell cycle distribution **(F)** Flow cytometric analysis of apoptosis **(G,I)** ALP staining (10×, scale bar = 500 μm) and quantification **(H,J)** Alizarin Red S (10×, scale bar = 500 μm) staining and quantification **(K)** RT-qPCR analysis of *Alp*, *Opg* and *Rankl* mRNA expression in MC3T3 cells **(L)** Volcano plot of differential gene expression (M–P) KEGG pathway and GO functional enrichment analyses **(Q–S)** GSEA enrichment analysis of oxidative stress, apoptosis, and osteogenic inhibition pathways. *p < 0.05, ***p < 0.001, ****p < 0.0001, n = 3. Unlabeled comparisons were not statistically significant.

The mRNA levels of the pro-inflammatory cytokines *IL-6* and *IL-17 R A* in MC3T3-E1 cells following exposure to ICM were assessed by RT-qPCR (Figures 8C,D). Treatment with ICM significantly upregulated the mRNA expression of *IL-6* and *IL-17 R A* compared with the control group, indicating that soluble factors secreted by iron-overloaded macrophages activate inflammatory signaling pathways in MC3T3-E1 cells. These findings were consistent with the increased concentrations of pro-inflammatory cytokines detected in the conditioned medium by ELISA. Notably, pretreatment with the ferroptosis inhibitor fer-1 or the iron chelator dfo partially attenuated the ICM-induced upregulation of *IL-6* and *IL-17 R A* mRNA levels.

Cell cycle analysis was performed, and the results are shown in Figure 8E. The control group exhibited a normal cell cycle distribution, with 51.76% of cells in G0/G1 phase, 35.69% in S phase, and 12.55% in G2/M phase. In the FAC-200 group, the proportion of cells in G0/G1 phase was significantly increased to 60.72%, accompanied by a reduction in S phase cells to 28.99%, which was consistent with the G0/G1 phase arrest induced by direct iron overload treatment in our previous experiments. In contrast, the ICM-treated group showed a marked decrease in G0/G1 phase cells to 44.46% and a substantial increase in S phase cells to 47.31%, indicating significant S phase cell cycle arrest. Notably, no significant differences in cell cycle distribution were observed between the ICM group and the ICM + fer-1 or ICM + dfo groups, suggesting that this cell cycle alteration is not affected by ferroptosis inhibition or iron chelation.

Apoptosis was analyzed *via* flow cytometry (Figure 8F). The apoptotic rate was 1.60% in the control group, and increased to 6.78% in the FAC-200 group, with early apoptosis as the predominant form. In contrast, the ICM group exhibited a robust pro-apoptotic effect, with a total apoptotic rate of 56.57%, 49.7% of which were late apoptotic or necrotic cells. The predominance of late apoptosis/necrosis in the ICM group, characterized by impaired cell membrane integrity and cytoplasmic leakage, was consistent with the lower ROS levels detected in the ICM group *versus* the FAC-200 group. Notably, the total apoptotic rates of the ICM + Fer-1 group (52.01%) and ICM + dfo group (54.40%) showed no significant difference from that of the ICM group (56.57%), indicating that neither intervention could reverse ICM-induced apoptosis.

To evaluate the effect of ICM on osteoblast differentiation, a systematic assessment was performed at both functional and molecular levels. After 7 days of osteogenic induction, ALP staining revealed a marked reduction in the purple-stained area in the ICM group compared with the control and FAC groups, which was only slightly increased by fer-1 or dfo pretreatment (Figure 8G). Quantitative analysis of ALP activity yielded consistent results, indicating that ICM exerts a stronger inhibitory effect on early osteogenic differentiation than iron overload alone (Figure 8I). Regarding late-stage differentiation, alizarin red S staining after 14 days of osteogenic induction showed that mineralized nodule formation was further reduced in the ICM group compared with the FAC group, and this inhibitory effect was partially reversed by fer-1 or dfo pretreatment, as confirmed by quantitative analysis (Figure 8H). RT-qPCR analysis demonstrated that ICM treatment significantly downregulated the mRNA expression (Figure 8K) of the key osteogenic gene *Alp*. Additionally, ICM markedly disrupted the RANKL/RANK/OPG axis, as evidenced by significant upregulation of Rankl and downregulation of *Opg*. Intervention with fer-1 or dfo significantly reversed the downregulation of *Alp* and *Opg* and suppressed the upregulation of *Rankl*. Notably, dfo exhibited a more pronounced inhibitory effect on *Rankl* expression, whereas fer-1 showed a greater capacity to restore *Opg* expression.

To elucidate the mechanism by which ICM affects MC3T3-E1 cells, transcriptome sequencing analysis was performed on MC3T3-E1 cells treated with or without ICM. Differential expression analysis revealed that 1,862 genes were significantly altered following ICM treatment compared with the control group, comprising 1,427 upregulated and 435 downregulated genes (Figure 8L). KEGG pathway enrichment analysis demonstrated that upregulated genes in the ICM group were primarily enriched in immune-and inflammation-related pathways, including the TNF signaling pathway, cytokine-cytokine receptor interaction, IL-17 signaling pathway, antigen processing and presentation, and NOD-like receptor signaling pathway. In contrast, downregulated genes were significantly enriched in pathways associated with cell proliferation and metabolism, such as cell cycle, DNA replication, ECM-receptor interaction, and PPAR signaling pathway (Figures 8M,N). GO functional enrichment analysis further corroborated these findings, showing that upregulated genes were mainly involved in biological processes including defense response to virus, immune response, inflammatory response, and cytokine activity, whereas downregulated genes were predominantly associated with cell cycle and proliferation-related functions such as DNA repair, DNA replication, and cell division (Figures 8O,P). GSEA confirmed that gene sets related to immune response and the IL-17 signaling pathway were significantly activated in the ICM group, while those associated with cell proliferation, including DNA replication, were markedly suppressed (Figures 8Q–S).

### 3.7 IL-17 signaling pathway mediates ICM-induced bone metabolic imbalance

Bone metabolism is a dynamic process coordinately regulated by bone formation and bone resorption. To systematically clarify the relationship between the IL-17 signaling pathway and immune-related osteoporosis, MC3T3-E1 cells were treated with ICM and an IL-17 signaling pathway antagonist to investigate its effects on osteogenesis-related functions. To determine the role of the IL-17 signaling pathway in ICM-induced cellular damage, the optimal concentration and treatment duration of the IL-17 pathway antagonist were first screened. As shown in Figure 9A,B, pretreatment with various concentrations of the antagonist for 1 h did not significantly affect cell viability in any group. After 6 h of pretreatment, 331 nM of the IL-17 pathway antagonist significantly reversed the inhibitory effect of ICM on cell viability (*p <* 0.01), whereas other concentrations showed no significant effect. Therefore, 331 nM IL-17 pathway antagonist with 6 h pretreatment was used as the IL-17 signaling blockade group in subsequent experiments. The effect of IL-17 signaling blockade on ICM-induced apoptosis was further evaluated. Apoptosis was detected by Annexin V/PI staining combined with flow cytometry, and the results are presented in Figure 9C. Compared with the control group, the apoptosis rate in the ICM group was significantly increased to 66.91% (*p <* 0.0001), of which late apoptosis accounted for 38.6%. In contrast, the apoptosis rate in the IL-17 signaling blockade group (IL-17AI) decreased to 32.85%, and the proportion of late apoptosis was reduced to 9.64%, indicating that inhibition of the IL-17 signaling pathway significantly attenuated ICM-induced cell death. To explore the role of the IL-17 signaling pathway in ICM-induced cell cycle disturbance, the effect of IL-17AI on cell cycle distribution was further analyzed. Flow cytometric results (Figure 9D) showed that the cell cycle distribution was normal in the control group. ICM treatment resulted in an abnormally increased proportion of cells in S phase and a significantly decreased proportion in G2/M phase, indicating severe cell cycle disturbance. After pretreatment with IL-17AI, the proportion of S phase cells was reduced, the G2/M phase proportion showed a recovery trend, and the cell cycle distribution was partially improved. These results demonstrated that blocking the IL-17 signaling pathway could partially alleviate ICM-induced cell cycle disorders.

**FIGURE 9:**
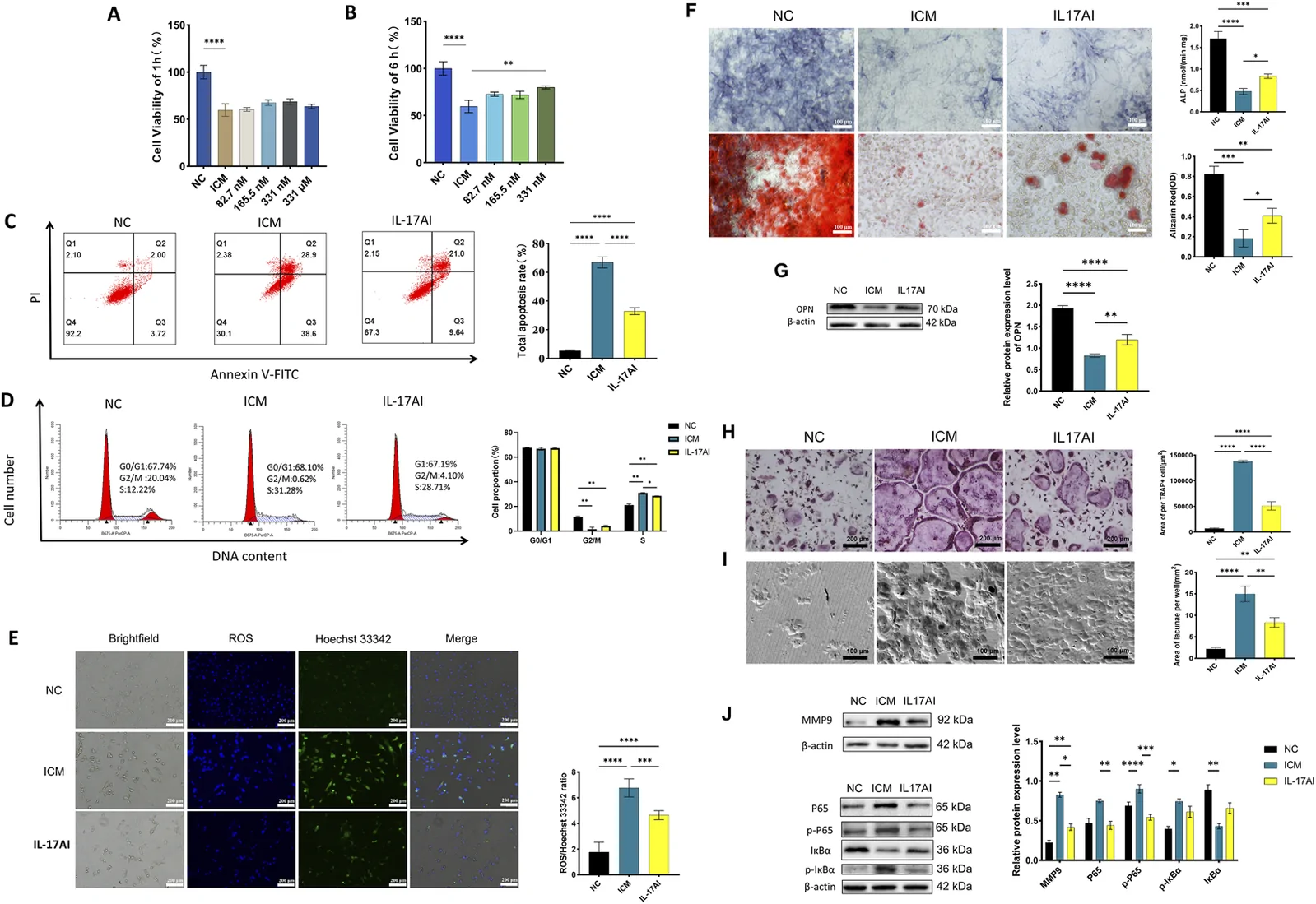
IL-17 pathway antagonist reverses ICM-induced dysfunction in MC3T3-E1 cells **(A,B)** CCK-8 assay of cell viability **(C)** Flow cytometric analysis of apoptosis **(D)** Flow cytometric analysis of cell cycle distribution **(E)** ROS fluorescence staining (10×, scale bar = 200 μm) and quantification **(F)** ALP and Alizarin Red S staining (20×, scale bar = 100 μm) and quantification **(G)** Western blot analysis of OPN expression **(H,I)** TRAP staining, scanning electron microscopy, and quantification of osteoclast number and bone resorption area **(J)** Western blot analysis of MMP9, CTSK, p65, p-p65, IκBα, and p-IκBα expression. *p < 0.05, **p < 0.01, ***p < 0.001, ****p < 0.0001, n = 3. Unlabeled comparisons were not statistically significant.

To investigate the role of the IL-17 signaling pathway in ICM-induced oxidative stress in MC3T3-E1 cells, intracellular ROS levels were detected using the DCFH-DA fluorescent probe (Figure 9E). Fluorescence images showed that only weak green fluorescence was observed in the blank control group, with intracellular ROS maintained at a basal level. In contrast, the ICM group exhibited bright green fluorescence with intense signals in the cytoplasm, indicating that ICM significantly induced ROS production in MC3T3-E1 cells. The green fluorescence intensity was markedly reduced in the IL-17AI pretreatment group, suggesting that blocking IL-17 signaling effectively attenuated ICM-induced ROS generation. Quantitative analysis using a fluorescence microplate reader showed that the ROS/Hoechst 33,342 ratio was 1.77 in the blank control group and increased to 6.77 in the ICM group, representing a 2.82-fold increase (*p <* 0.0001), confirming that ICM significantly aggravated intracellular oxidative stress by activating proinflammatory signaling pathways. Following IL-17AI pretreatment, the ratio decreased to 4.64, a 31.4% reduction compared with the ICM group (*p <* 0.001). Although IL-17AI did not fully restore ROS levels to those of the control group, its mitigating effect on oxidative stress was statistically significant. These results indicate that the IL-17 signaling pathway plays a critical pro-oxidant role in ICM-induced oxidative stress in MC3T3-E1 cells, and blocking this pathway significantly alleviates cellular oxidative damage.

To determine the protective effect of IL-17 signaling blockade on osteogenic function, its effects on cell differentiation and mineralization were further evaluated (Figure 9F). ALP staining and quantitative activity analysis after 7 days of osteogenic induction showed that ALP activity was significantly decreased in the ICM group compared with the control group (*p <* 0.001), and IL-17AI pretreatment significantly reversed this inhibitory effect (*p <* 0.05). Alizarin Red S staining and quantitative analysis after 14 days of osteogenic induction revealed that mineralized nodule formation was markedly reduced in the ICM group (*p <* 0.0001), and IL-17AI intervention significantly promoted mineralized nodule deposition (*p <* 0.05). At the protein level, Western blot analysis (Figure 9G) demonstrated that ICM treatment significantly downregulated the expression of the key osteogenic protein OPN, which was notably reversed by IL-17AI pretreatment. These results indicated that activation of the IL-17 signaling pathway is involved in ICM-induced osteogenic dysfunction, and blocking this pathway effectively alleviates the impairment of osteogenic differentiation and mineralization caused by ICM.

To investigate the role of the IL-17 signaling pathway in ICM-regulated osteoclast differentiation and function, TRAP staining and bone resorption lacuna analysis were performed (Figure 9H). TRAP staining results showed that ICM treatment significantly promoted the fusion and maturation of osteoclast precursors, characterized by increased cell volume and more multinucleated cells; the average area of single cells was approximately 18.6-fold higher than that in the control group (*p <* 0.0001). In contrast, IL-17AI pretreatment effectively inhibited this effect, reducing the average area of osteoclasts by approximately 59.8% compared with the ICM group (*p <* 0.0001). Bone resorption lacuna experiments further confirmed (Figure 9I) that ICM treatment increased the bone resorption area by approximately 6.8-fold compared with the control group (*p <* 0.0001), indicating that it significantly enhanced the bone degradation activity of osteoclasts. After IL-17AI intervention, the bone resorption area was reduced by approximately 44.4% compared with the ICM group (*p <* 0.0001).

To elucidate the downstream mechanism of the IL-17 signaling pathway in regulating osteoclast function, the expression of key osteoclast proteins and NF-κB pathway-related molecules was detected by Western blot (Figure 9J). The results showed that ICM treatment significantly upregulated the expression of osteoclast functional proteins MMP9 and CTSK, and IL-17AI pretreatment effectively reversed this upregulation. In the present study, ICM treatment activated the canonical NF-κB signaling pathway, as evidenced by reduced expression of the inhibitory protein IκBα, alongside upregulation of total P65 transcription factor subunit and significant increases in both p-IκBα and p-P65. After IL-17AI pretreatment, the expression of p-IκBα, P65, p-P65 were markedly inhibited, while the protein level of IκBα was partially restored (Figure 9J). These results suggest that ICM may activate the IL-17 signaling to promote NF-κB pathway activation, thereby upregulating the expression of osteoclast-related proteins such as MMP9 and CTSK, and ultimately enhancing osteoclast differentiation and bone resorption. Blocking IL-17 signaling effectively suppresses this cascade.

## 4 Discussion

In this study, we systematically investigated the dual pathological mechanisms by which iron overload disrupts bone homeostasis, integrating *in vivo* and *in vitro* approaches to explore the potential etiology of iron-associated bone loss. Our findings reveal that iron overload induced skeletal deterioration not merely through a singular toxic effect, but through two synergistic pathological pathways that converge to compromise bone mass and microarchitecture. Specifically, we identified a direct ferroptosis-mediated osteogenic impairment pathway, wherein intracellular iron accumulation triggers lipid peroxidation and osteoblast dysfunction, alongside an indirect IL-17-driven inflammatory immune pathway, characterized by macrophage polarization and the secretion of pro-inflammatory cytokines that further suppress bone formation. These distinct yet interconnected mechanisms were illustrated in Figure 10.

**FIGURE 10:**
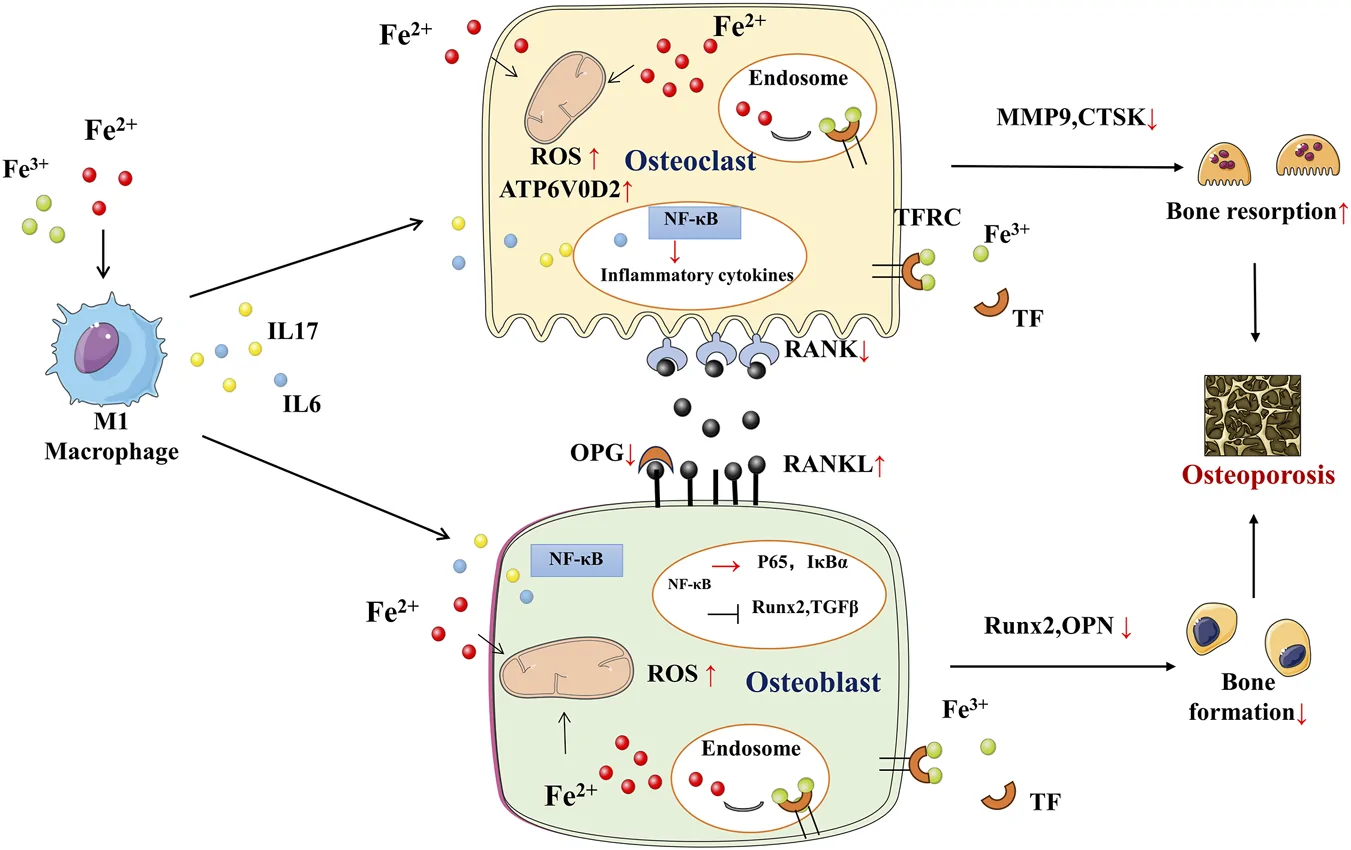
Schematic diagram of the dual mechanisms underlying osteoporosis induced by iron overload.

On one hand, extracellular Fe^3+^ enters cells and is reduced to Fe^2+^ in endosomes, which generates hydroxyl radicals *via* the Fenton reaction and directly triggers lipid peroxidation and ferroptosis. Ferroptosis is a form of programmed cell death distinct from apoptosis and necrosis, characterized by iron-dependent lipid peroxide accumulation and glutathione depletion (Guggisberg et al., 2022; Guggisberg and Ryu, 2019). In the present study, iron overload induced classic ferroptotic features in MC3T3-E1, including increased intracellular Fe^2+^ and MDA levels, decreased GSH content, and downregulation of the anti-ferroptotic proteins GPX4 and SLC7A11. These changes were accompanied by mitochondrial shrinkage, increased membrane density, and cristae reduction—morphological hallmarks of ferroptosis (Jiang et al., 2024). Importantly, treatment with the ferroptosis inhibitor fer-1 or the iron chelator dfo significantly reversed these alterations, confirming that ferroptosis is a key mechanism underlying iron overload-induced osteoblast dysfunction. Notably, transcriptomic analysis identified *Atp6v0d2*, a subunit of the V-ATPase complex, as significantly upregulated under iron overload. Although traditionally considered an osteoclast-specific marker, emerging evidence has demonstrated its expression and functional roles in non-osteoclast lineages (Jähn et al., 2017). In the present study, *Atp6v0d2* was specifically upregulated in iron-overloaded MC3T3-E1, and this upregulation was reversed by fer-1 or dfo. Functional knockdown of *Atp6v0d2* not only alleviated ferroptosis and rescued osteogenic differentiation but also suppressed osteoclast differentiation and bone resorption activity. Mechanistically, aberrant upregulation of *Atp6v0d2* may exacerbate lysosomal iron homeostasis imbalance, synergize with the RANKL-RANK axis to enhance osteoclast function (Yang et al., 2019), and directly inhibit osteoblast differentiation *via* oxidative stress. These findings positioned *Atp6v0d2* upregulation as a central hub in iron overload-induced bone homeostasis disruption.

Macrophages are key regulators of skeletal homeostasis, capable of polarizing toward a pro-inflammatory M1 or reparative M2 phenotype in response to microenvironmental signals (Batoon et al., 2021; Meng et al., 2023). In the present study, iron overload dose-dependently promoted M1 polarization of RAW264.7 macrophages, as evidenced by an increased CD86^+^/CD206^+^ cell ratio, upregulation of M1-associated marker genes (CD86, Inos, IL-6, Tnf), and elevated secretion of pro-inflammatory cytokines (TNF-α, IL-6), accompanied by reduced M2 markers and anti-inflammatory cytokine levels. These findings suggest that macrophages in the iron-overloaded microenvironment shift toward a pro-inflammatory phenotype and may influence neighboring osteoblast function through the secretion of soluble factors. To test this hypothesis, we treated MC3T3-E1 osteoblasts with conditioned medium from iron-overloaded macrophages (ICM). ICM markedly inhibited osteoblast viability, induced S-phase cell cycle arrest, and promoted apoptosis. These effects were not reversed by the ferroptosis inhibitor Fer-1 or the iron chelator DFO, indicating that the mechanism of ICM-induced injury is distinct from direct iron toxicity. To further elucidate the molecular basis of ICM-induced osteoblast injury, transcriptomic analysis was performed on ICM-treated MC3T3-E1 cells. The results showed significant enrichment of immune- and inflammation-related pathways, with the IL-17 signaling pathway being particularly prominent. GSEA further demonstrated that this pathway exhibited the highest enrichment significance among all inflammation-related pathways, and was therefore prioritized for subsequent investigation. From a functional perspective, IL-17 is a well-recognized “bridge molecule” linking immune inflammation and bone metabolism. It can directly promote osteoclast differentiation and bone resorption by inducing RANKL expression in osteoblasts (Shukla et al., 2018), while simultaneously suppressing osteoblast function, and has been established as a core pathogenic mediator in various bone loss diseases (He et al., 2021). In contrast, TNF-α, as an upstream broad-spectrum inflammatory factor, exerts its regulatory effects on bone metabolism largely through indirect induction of downstream effectors; the NOD-like receptor pathway primarily participates in innate immune recognition and acts upstream of the immune response; and the cytokine-cytokine receptor interaction pathway represents a generalized interaction network lacking bone-specific effects. Thus, based on both enrichment significance and functional specificity, the IL-17 pathway was selected for further mechanistic investigation. To systematically evaluate the activation status and functional output of this pathway in osteoblasts, we examined the mRNA expression of IL-17 R A and IL-6. IL-17 R A encodes the functional receptor subunit of the IL-17 signaling pathway and represents the upstream component; its expression changes in osteoblasts can directly reflect whether the responsiveness of osteoblasts to IL-17 signaling is regulated by the inflammatory microenvironment. IL-6, a classic pro-inflammatory cytokine, serves as a typical downstream effector following IL-17 pathway activation and a sensitive indicator of the inflammatory response in osteoblasts (Tokuda et al., 2004). Thus, the combined detection of IL-17 R A and IL-6 reflects the upstream receptor status and downstream effector output of this pathway, respectively, constituting a complete validation framework from signal reception to functional response. Upon binding to IL-17 R A, IL-17 activates downstream cascades including the NF-κB pathway, which on one hand promotes osteoclast differentiation by inducing RANKL expression (Zhu et al., 2023; Mios et al., 2012), and on the other hand suppresses osteoblast function through oxidative stress and cell cycle arrest (Zhang et al., 2022; Xiao et al., 2017). In this study, ICM activated the canonical NF-κB pathway in osteoblasts, and blockade of IL-17 signaling not only reversed this activation but also ameliorated both osteoblast injury and osteoclast overactivation, suggesting that the IL-17-NF-κB axis is a key downstream effector pathway mediating ICM-induced bone metabolic imbalance. Collectively, these results confirm that IL-17 signaling serves as a critical bridge linking iron overload-induced inflammation to bone metabolic imbalance.

The findings of the present study suggest that the *Atp6v0d2* and IL-17 signaling pathways play important roles in iron overload-induced osteoporosis. Targeting *Atp6v0d2* may attenuate ferroptosis in osteoblasts while simultaneously suppressing excessive osteoclast activation, whereas blocking IL-17 signaling holds promise for correcting both aberrant bone resorption and impaired bone formation. This dual-target strategy may offer a more effective therapeutic approach for iron overload-related osteoporosis. Nevertheless, the current study remains at the level of functional validation, and several limitations should be acknowledged. First, the precise molecular mechanisms by which Atp6v0d2 regulates lysosomal iron homeostasis and ferroptosis have yet to be fully elucidated. To date, our conclusions are primarily based on gene knockdown experiments, lacking complementary validation through *Atp6v0d2* overexpression, as well as direct experimental evidence including dynamic lysosomal pH monitoring and ferritinophagy flux analysis. Moreover, the causal relationship between *Atp6v0d2*-mediated lysosomal acidification and intracellular labile iron release has not been directly established. In addition, all osteoblast-related mechanistic experiments were performed using the MC3T3-E1 preosteoblast cell line rather than primary osteoblasts, which may limit the physiological relevance of our findings to native osteoblast populations *in vivo*. Second, the upstream regulators of the IL-17 signaling pathway and its crosstalk with the RANKL/OPG axis within the iron-overloaded bone microenvironment warrant further investigation. Furthermore, although the pro-apoptotic effect of the iron overload-induced inflammatory microenvironment on osteoblasts has been validated *in vitro*, direct confirmation of apoptotic osteoblast identity in bone tissue *via in vivo* immunofluorescence co-localization staining is still lacking. Third, while the core pathological phenotypes of iron overload have been confirmed *in vivo*, the translational potential of targeting *Atp6v0d2* and IL-17 remains to be demonstrated through *in vivo* gene editing or targeted pharmacological intervention studies in animal models once the molecular mechanisms are clarified. Additionally, our iron overload rat model was established by acute administration of FAC, which differs from the chronic iron accumulation observed in clinical scenarios such as hereditary hemochromatosis and long-term transfusion-dependent conditions. Moreover, only male rats were included in the *in vivo* experiments, leaving the potential interactions between estrogen and iron metabolism, as well as sex-related differences in bone homeostasis regulation, unexplored-factors that may limit the generalizability of our current conclusions. In future studies, single-cell RNA sequencing will be conducted to characterize the heterogeneous responses of bone cell subpopulations to iron overload and IL-17 signaling, combined with conditional gene knockout mouse models to define the *in vivo* roles of these pathways. Concurrently, lysosomal functional probes and dynamic iron metabolism monitoring techniques can be applied to dissect the molecular cascade through which *Atp6v0d2* regulates ferroptosis. Furthermore, given the crosstalk between IL-17 signaling and iron overload in regulating *Atp6v0d2* expression, exploring the regulatory network governing their interplay may provide a theoretical basis for developing combination therapeutic strategies that simultaneously target both pathways. Nonetheless, our findings regarding iron overload-mediated ferroptosis and immune dysregulation may offer novel insights into the pathogenesis of osteoporosis.

## 5 Conclusion

In conclusion, this study demonstrated that iron overload induces ferroptosis and inflammatory responses in Male Sprague-Dawley rats, thereby disrupting bone metabolic homeostasis. At the cellular level, iron overload triggered ferroptosis in MC3T3-E1 cells and enhanced osteoclast activity. *Atp6v0d2* was identified as a key ferroptosis-related gene; its knockdown inhibited osteoclast differentiation and partially restored osteogenic function. Additionally, iron overload promoted M1 macrophage polarization and the release of inflammatory factors, forming a microenvironment that exacerbates bone injury, and blockade of the IL-17 pathway reversed these effects. This study proposes a dual-pathway model of iron overload-induced osteoporosis: direct ferroptosis induction and indirect inflammatory disturbance, with *Atp6v0d2* and IL-17 signaling as core mediators. These findings provide novel insights into the pathogenesis of iron overload-related osteoporosis and offer a new perspective for understanding bone metabolism dysregulation under iron overload conditions.

## Data Availability

The original contributions presented in the study are publicly available. This data can be found here: NCBI Sequence Read Archive, BioProject accession PRJNA1537712.
