# Supplementary material for: Iron overload disrupts bone homeostasis via dual pathways: inducing ferroptosis of preosteoblastic cells and shaping a proinflammatory immune milieu
Source: Front Pharmacol. 2026 Oct 16;17:1905654. doi: 10.3389/fphar.2026.1905654 (PMC13719617; doi:10.3389/fphar.2026.1905654)
Supplement: Supplementary file 1 [file Table1.DOCX]

| Gene | | Primer Sequence（5’‒3’） | | Length（bp） |
| --- | --- | --- | --- | --- |
| *Gapdh* | F: GCACAGTCAAGGCCGAGAAT | | 20 | |
|  | R: GCCTTCTCCATGGTGGTGAA | | 20 | |
| *Ocn* | F: CAGACCTAGCAGACACCATGAG | | 25 | |
|  | R: CGTCCATACTTTCGAGGCAG | | 20 | |
| *Grx* | F: TTCCATGCTGCGATCCTACC | | 20 | |
|  | R: AGCTTCTCTTAGGCCGTTCCT | | 21 | |
| *Col1a1* | F: GCTCCTCTTAGGGGCCACT | | 19 | |
|  | R: ATTGGGGACCCTTAGGCCAT | | 20 | |
| *Alp* | F: CCAACTCTTTTGTGCCAGAGA | | 21 | |
|  | R: GGCTACATTGGTGTTGAGCTTTT | | 23 | |
| *Gpx4* | F: TGTGCATCCCGCGATGATT | | 19 | |
|  | R: CCCTGTACTTATCCAGGCAGA | | 21 | |
| *Opg* | F: CCTTGCCCTGACCACTCTTAT | | 21 | |
|  | R: CACACACTCGGTTGTGGGT | | 19 | |
| *Rankl* | F: AGCCGAGACTACGGCAAGTA | | 20 | |
|  | R: AAAGTACAGGAACAGAGCGATG | | 22 | |
| *Lars* | F: ATGGACCTCACAGTAACTCACA | | 22 | |
|  | R: ACCATAGCAATCTTTGCCTTCA | | 22 | |
| *Sirt1* | F: TGGACAATTCCAGCCATCTC | | 20 | |
|  | R: GCGTGTCTATGTTCTGGGTATAG | | 23 | |
| *Pla2g2e* | TCCTGGATACTGCTCCTACTACT | | 23 | |
|  | GACCATTCCTCATTGCACACA | | 21 | |
| *Fndc1* | F: CAGTAAGGCGGATGTTGAGC | | 20 | |
|  | R: TTGGGGAGAAGCAGGCAC | | 18 | |
| *Slc7a11* | F: GGCACCGTCATCGGATCAG | | 19 | |
|  | R: CTCCACAGGCAGACCAGAAAA | | 21 | |
| *Atp6v0d2* | F: CAGACCTAGCAGACACCATGAG | | 22 | |
|  | R: CGTCCATACTTTCGAGGCAG | | 20 | |
| *Arg2* | F: ACGGGCAAATTCCTTGCGT | | 19 | |
|  | R: TGGACCATATTCCACTCCTAGC | | 22 | |
| *Grx* | F: GGCTGCTGCTAAGATCAAGA | | 20 | |
|  | R: TGATGGTCTTGGCAAAGATG | | 20 | |
| *Atp13a4* | F: GCAAACTGTGTCCCATGTTCC | | 21 | |
|  | R: CCTCGTCTGTAATGAGAGGGT | | 21 | |
| *Mmp3* | F: TATGGACCTCCCCCTGACTCC | | 20 | |
|  | R: CAGGTTCAAGCTTCCTGAGG | | 20 | |
| *Inos* | F: GTTCTCAGCCCAACAATACAAGA | | 23 | |
|  | R: GTGGACGGGTCGATGTCAC | | 19 | |
| *Tgf-β* | F: CCACCTGCAAGACCATCGAC | | 20 | |
|  | R: CTGGCGAGCCTTAGTTTGGAC | | 21 | |
| *Arg1* | F: CCAGGAGAGGCATTATGAGCA | | 21 | |
|  | R: ACTGTCGGAGGTAGGAGTGC | | 20 | |
| *IL10* | F: CTTACTGACTGGCATGAGGATCA | | 23 | |
|  | R: GCAGCTCTAGGAGCATGTGG | | 20 | |
| *CD86* | F: TCAATGGGACTGCATATCTGCC | | 20 | |
|  | R: GCCAAAATACTACCAGCTCACT | | 22 | |
| *TNF-α* | F:CAGGCGGTGCCTATGTCTC | | 19 | |
|  | R:CGATCACCCCGAAGTTCAGTAG | | 22 | |
| *IL-17ra* | F: CGGAGAATTAGTCCCTGTGTTG | | 22 | |
|  | R: GAACAGTCACTTCATACTCCTGG | | 23 | |
| *IL6* | F: CTGCAAGAGACTTCCATCCAG | | 21 | |
|  | R: AGTGGTATAGACAGGTCTGTTGG | | 23 | |
| *CD206* | F: CTCTGTTCAGCTATTGGACGC | | 21 | |
|  | R: TGGCACTCCCAAACATAATTTGA | | 22 | |

Table S1. Primer sequences used for RT-qPCR

Fig. S1. Quality control analysis of the RNA-sequencing dataset. (A) Principal component analysis of transcriptome profiles. (B) Pearson correlation matrix of all biological replicates.
